# The deubiquitinase USP36 Regulates DNA replication stress and confers therapeutic resistance through PrimPol stabilization

**DOI:** 10.1093/nar/gkaa1090

**Published:** 2020-11-25

**Authors:** Yuanliang Yan, Zhijie Xu, Jinzhou Huang, Guijie Guo, Ming Gao, Wootae Kim, Xiangyu Zeng, Jake A Kloeber, Qian Zhu, Fei Zhao, Kuntian Luo, Zhenkun Lou

**Affiliations:** Department of Pharmacy, Xiangya Hospital, Central South University, Changsha 410008, Hunan, China; Department of Oncology, Mayo Clinic, Rochester, MN 55905, USA; Department of Pathology, National Clinical Research Center for Geriatric Disorders, Xiangya Hospital, Central South University, Changsha 410008, Hunan, China; Department of Oncology, Mayo Clinic, Rochester, MN 55905, USA; Department of Oncology, Mayo Clinic, Rochester, MN 55905, USA; Department of Oncology, Mayo Clinic, Rochester, MN 55905, USA; Department of Oncology, Mayo Clinic, Rochester, MN 55905, USA; Department of Oncology, Mayo Clinic, Rochester, MN 55905, USA; Department of Oncology, Mayo Clinic, Rochester, MN 55905, USA; Mayo Clinic Medical Scientist Training Program, Mayo Clinic, Rochester, MN 55905, USA; Department of Molecular Pharmacology and Experimental Therapeutics, Mayo Clinic, Rochester, MN 55905, USA; Department of Oncology, Mayo Clinic, Rochester, MN 55905, USA; Department of Oncology, Mayo Clinic, Rochester, MN 55905, USA; Department of Oncology, Mayo Clinic, Rochester, MN 55905, USA; Department of Oncology, Mayo Clinic, Rochester, MN 55905, USA

## Abstract

PrimPol has been recently identified as a DNA damage tolerant polymerase that plays an important role in replication stress response. However, the regulatory mechanisms of PrimPol are not well defined. In this study, we identify that the deubiquitinase USP36 interferes with degradation of PrimPol to regulate the replication stress response. Mechanistically, USP36 is deubiquitinated following DNA replication stress, which in turn facilitates its upregulation and interaction with PrimPol. USP36 deubiquitinates K29-linked polyubiquitination of PrimPol and increases its protein stability. Depletion of USP36 results in replication stress-related defects and elevates cell sensitivity to DNA-damage agents, such as cisplatin and olaparib. Moreover, USP36 expression positively correlates with the level of PrimPol protein and poor prognosis in patient samples. These findings indicate that the regulation of PrimPol K29-linked ubiquitination by USP36 plays a critical role in DNA replication stress and chemotherapy response.

## INTRODUCTION

DNA polymerases are enzymes that synthesize DNA during genome duplication, and are critical for protecting cells against DNA damage. Cancer cells universally display uninhibited DNA replication; therefore, DNA polymerases have been suggested as potential therapeutic targets to combat multiple types of cancer (1,2). Recently, a new DNA damage tolerant polymerase, called PrimPol, has the ability to catalyze both primase and DNA polymerase reactions (3). The importance of PrimPol is underscored by the finding that PrimPol-mediated adaptive responses might decrease the efficiency of anti-cancer genotoxic drugs (4). Moreover, it is important to note that overexpression of PrimPol is correlated with prolonged survival of a subset of cancer cells, most likely by promoting the replication progression (5,6). Therefore, the study of the PrimPol and its regulatory network would not only reveal how PrimPol coordinates the dynamic reactions in DNA replication, but also provide novel targets for therapeutic intervention in cancers.

PrimPol has been recently characterized to initiate nuclear and mitochondrial uninterrupted fork progression following DNA replication stress (3,7). Functional interaction indicated that proliferating cell nuclear antigen (PCNA) is required for relocation of PrimPol to stalled replication forks, and mediates the activity of both replicative and translesion DNA synthesis (4,8). In a large-scale pull-down assay, polymerase delta-interacting protein 2 (PolDIP2) was discovered as a potential cellular binding partner of PrimPol (9). It was reported that PolDIP2 is required for the DNA damage tolerance, specifically through stimulating the DNA polymerase activity of PrimPol. PolDIP2 also interacts with PCNA, together to regulate DNA polymerase activity of PrimPol (9,10). Another study demonstrated that replication protein A (RPA) binds PrimPol, and is required for relocating PrimPol onto sites of DNA damage (11). Like other critical factors involved in DNA replication, PrimPol activity and expression need to be tightly regulated. However, it is still unclear how PrimPol is dynamically regulated.

It is well known that ubiquitination plays an important regulatory role in the response to DNA replication stress (12,13). However, whether PrimPol is also directly regulated by a deubiquitinating enzyme (DUB) is unknown and deserves further investigation. In this study, we identified a deubiquitinase, USP36, that regulates PrimPol ubiquitination *in vitro* and in cells. In addition, USP36 regulates cancer therapeutic resistance in a PrimPol-dependent manner. In ovarian cancer tissues, USP36 overexpression is observed to positively correlate with the level of PrimPol expression and poor prognosis, indicating that the USP36-PrimPol axis may play an important regulatory mechanism in the cancer pathogenesis and treatment.

## MATERIALS AND METHODS

### Cell culture and inhibitor

HEK293T (CRL-3216) cell lines were purchased from ATCC. Human ovarian cancer cell lines A2780 was kindly provided by Dr Scott Kaufmann (Mayo Clinic). OVCAR8 (CVCL-1629), SK-OV-3 (HTB-77), OV-90 (CRL-11732), OVCAR10 (CVCL-4377), IGROV1 (CVCL-1304) were purchased from ATCC. OVKATE (CVCL-3110) was from serious papillary adenocarcinomas (14). OV-56 (96020759) and PEO1 (10032308) were purchased from Sigma. The identities of all cell lines were confirmed by the Medical Genome Facility at Mayo Clinic Center (Rochester, MN) using short tandem repeat profiling upon receipt. Cell lines were cultured in Dulbecco's Modified Eagle's Medium or McCoy's 5A supplemented with 10% fetal bovine serum (FBS) at 37°C in 5% (v/v) CO_2_. The following inhibitors were used: MG132 (Selleckchem: S2619), Cycloheximide (Sigma: 01810).

### Plasmids and antibodies

Myc-PrimPol WT vector was kindly provided by Dr Jun Huang (15). All Flag-tagged USP36 truncated mutants (WT, 1–420, 421–800, 1–800 and 801–1121), V5-tagged USP36 (WT and C131A mutant) and GST-tagged USP36^1-800^ (WT and C131A mutant) plasmids were kindly provided by Dr Mushui Dai (16). Flag-USP36-WT-K329R, Flag-USP36-WT-K338R, Flag-USP36-WT-K329R/K338R (2KR) were generated by site-directed mutagenesis according to standard protocol. c-Myc shRNA (#15662) was described previously (17,18) and obtained from Addgene. The plasmid pRK5-HA-Ubiquitin-K29R (#17602) (19) was purchased from Addgene. The following antibodies were used: anti-PrimPol (Novus Biologicals: NBP2-67217, 1:750), anti-USP36 (Proteintech: 14783-1-AP, 1:500), anti-Ubiquitin (Santa Cruz: sc8017, 1:1000), anti-GAPDH (Proteintech: 60004-1-lg, 1:10,000), anti-HA (Sigma: H6908, 1:1000), anti-Flag (Sigma: F1804, 1:1000), anti-V5 (Invitrogen: R960, 1:1000), anti-Myc (Cell Signaling Technology: 2276S, 1:1000), anti-VDAC1 (Cell Signaling Technology: 4866T, 1:1000), anti-BrdU antibody (BD Bioscience: 347580, 1:100; Abcam: ab6326, 1:1000), Alexa Fluor 594 anti-Rat IgG antibody (Invitrogen: A-11007, 1:1000), Alexa Fluor 488 anti-Mouse IgG antibody (Jackson ImmunoResearch: 115-545-062, 1:1000), Rhodamine Red™-X anti-Mouse IgG (H+L) antibody (Jackson ImmunoResearch: 115-295-146, 1:1000), Alexa Fluor 488 anti-Rabbit IgG antibody (Jackson ImmunoResearch: 111-545-045, 1:1000), anti-H3 (Cell Signaling Technology: 4620S, 1:10 000), anti-Fibrillarin (Santa Cruz: sc166001, 1:100), γH2AX (Millipore: 05-636, 1:500).

### Lentiviruses

All USP36 and Pirmpol shRNAs were purchased from Sigma. USP36 shRNA-1: CCGGGCGGTCAGTCAGGATGCTATTCTCGAGAATAGCATCCTGACTGACCGCTTTTT (TRCN0000022447), USP36 shRNA-2: CCGGGCGACTGAAT ATACACCTGTACTCGAGTACAGGTGTATATTCAGTCGCTTTTT (TRCN0000022444). PrimPol shRNAs: CCGGGCTCCATGATGTGGCATTTAA CTCGAGTTAAATGCCACATCATGGAGCTTTTTTG (TRCN0000135447), CCG GGTCAGGTTCTCAGATACTTTACTCGAGTAAAGTATCTGAGAACCTGACTTTTTTG (TRCN0000134668). TransIT-X2 (MIRUS Bio) was used to co-transfect the shRNA vector, the envelope plasmid pMD2.G, and the packaging plasmid psPAX2 into HEK293T cells. Media containing lentivirus was collected 48 h after transfection. The viruses were then used to infect cells in the presence of polybrene (8 μg/ml). The cells were harvested at 48 h posttransduction for immunoblotting analysis.

### Immunoprecipitation and immunoblotting

For immunoprecipitation, the cells were lysed with NETN lysis buffer (20 mM Tris–HCl, pH 8.0, 500 mM NaCl, 1 mM EDTA and 0.5% NP-40) supplemented with protease inhibitors for 15 min. After centrifugation at 12 000 rpm for about 15 min, the soluble fractions were collected. Cell lysates were incubated with antibody and Agarose beads (Thermo Scientific) for overnight at 4°C. After incubation, the Agarose beads were washed three times with NETN buffer, eluted with 4× SDS loading buffer, and resolved on SDS-PAGE. Then, PVDF membranes (Millipore) were blocked in 5% skim milk in TBST buffer and immunoblotted with antibodies as indicated. For immunoblotting, cells were lysed in NETN lysis buffer supplemented with protease inhibitors for 15 min. After centrifugation at 12,000 rpm for about 15 min, the soluble fractions were collected. The Supernatants were boiled in 4× SDS loading buffer at 95°C for about 10 min, and then resolved on SDS-PAGE and immunoblotted with with antibodies as indicated. Western blots were analyzed using the Tanon 5200 imaging system.

### Preparation of mitochondria fraction

Mitochondrial extracts were isolated according to the manufacturer's instructions (Thermo Scientific, 89874). In brief, 2 × 10^7^ cells were suspended with 800 μl of Mitochondria Isolation Reagent A for 2 min, and then 10 μl of Mitochondria Isolation Reagent B was added for 5 min. Subsequently, 800 μl of Mitochondria Isolation Reagent C was added to the cell suspension. The supernatant was centrifuged at 12 000 g for 15 min at 4°C. The pellet (mitochondria) was rinsed with 500 μl of Mitochondria Isolation Reagent C and recentrifuged at 12 000 g for 5 min. Then, the pellets of mitochondria were lysed with 100 μl of 2% CHAPS in TBS. After centrifugation at high speed for 2 min, the supernatant containing mitochondrial proteins can be separated by SDS-PAGE and immunoblotted with indicated antibodies.

### Deubiquitination *in vivo* and *in vitro*

The *in vivo* and *in vitro* deubiquitination assays were performed as described previously (20). USP36 WT and CA mutant and Myc-PrimPol were overexpressed in HEK293T cells. After treatment with MG132, cells were lysed in the SDS buffer and boiled for 10 min. After diluting with NETN buffer (1:10 ratio) containing 1 mM iodoacetamide and 20 mM NEM, cell lysates were immunoprecipitated with anti-Myc beads for overnight at 4°C. Precipitates were washed with NETN about 4 times and were immunoblotted as described above. For the *in vitro* assay, His-Ub conjugated Myc-PrimPol was purified from HEK293T cells through multiple steps as previously described (20). The purified GST-tagged USP36^1–800^ (WT and C131A mutant) was incubated with Ub-Myc-PrimPol in deubiquitination buffer for overnight at 16°C

### Quantitative real time-PCR analysis

Total RNA was isolated from cells using Trizol (Invitrogen, #15596026). Reverse transcriptions were performed with High-Capacity cDNA Reverse Transcription Kit (Invitrogen, #4368813). A quantitative Real Time-PCR (qRT-PCR) was developed on an ABI StepOne real-time PCR system (Applied Biosystems) using Power SYBR™ Green PCR Master Mix (Bio-Rad, #4367659). All reactions were carried out in triplicate. Relative gene expression was calculated using the ΔC_t_ method following the manufacturer's instruction. The primers for PrimPol are 5′-TTACCTTGTGACAACCTATGCTG-3′ and 5′-ACACCGTATAACTCTTGAAGTGC-3′. And the primers for USP36 are 5′-AGGGAGCTAGTCGCCACAA-3′ and 5′-GTGTGTAGGTCAAGCACTGGA-3′.

### Colony formation assay

To measure cell survival in the presence of cisplatin, olaparib, ultraviolet irradiation (UV), or hydroxyurea (HU), 800 cells were plated in triplicate in each well of six-well plates. After 16 h, cells were treated with the indicated concentrations of the DNA damaging agents, and left for 10–14 days at 37°C to allow colony formation. Colonies were fixed with 100% ethanol, stained with 0.006% crystal violet solution (0.006% crystal violet, 25% methanol), and colonies were counted. Results were normalized to plating efficiencies.

### DNA fiber assay

To check restart efficiency of stalled replication forks, cells were pulse-labeled with 5-lodo-2′-deoxyuridine (IdU) and 5-chloro-2′-deoxyuridine (CIdU) as previously reported (21). After first labeling with ldU 25 μM for 20 min, cells were washed twice with media. Cells were then washed and treated with HU 4 mM for 2 h. After additional washes with media, cells were recovered in fresh medium containing CIdU 200 μM for 1 h. Labeled cells were harvested and resuspended in 150 μl of lysis buffer (200 mM Tris–HCl, pH 7.4, 50 mM EDTA and 0.5% SDS) on a clean glass slide. For immunodetection of labeled tracks, fibers were incubated with primary antibodies at 4°C for overnight and the corresponding secondary antibodies for 1 h at room temperature, in a humidity chamber. Fiber images were visualized using a Nikon eclipse 80i Fluorescence microscope. All fiber lengths were measured and analyzed using Image J software.

### Immunofluorescence and in situ PLA assay

For γH2AX immunofluorescence assays, cells were spun onto glass slides and stained as described (22). For USP36 immunofluorescence, cells were permeabilized in 0.5% Triton-X solution for 5 min at room temperature, and then fixed with 3% paraformaldehyde for 15 min. The samples were incubated with primary antibody for 1 h at 37°C, washed three times with TBST to remove the non-specific binding, incubated with the secondary antibody for about 30 min. Cells were then stained with DAPI to visualize nuclear DNA. The in situ PLA assay was carried out using a Duolink in situ PLA kit (Sigma, # DUO92101) according to the manufacturer's instructions. Cells grown on cover glass were washed with PBS twice and incubated in 3% paraformaldehyde for 15 min, and permeabilized in 0.5% Triton-X solution for 5 min at room temperature. After blocking, samples were incubated with USP36 and PrimPol antibodies for 1 h. Samples were washed twice times and incubated with Duolink PLA Probe for 1 h at 37°C, Duolink Ligation buffer for 30 min at 37°C and Amplification buffer for 100 min at 37°C. Cells were then stained with DAPI to visualize nuclear DNA. The coverslips were mounted onto glass slides with anti-fade solution and visualized using a Nikon eclipse 80i fluorescence microscope. Counting of the PLA signal dots was done using ImageJ.

### Isolation of proteins on nascent DNA (iPOND)

iPOND assay was performed as previously described (23). In brief, after first labeling with EdU 10 μM for 10 min, cells were washed three times with the washing buffer. Cells were then treated with HU 10 mM for 4 h. Following this, cells were fixed with 1% formaldehyde solution for 20 min at room temperature. Formaldehyde was quenched by adding 1 ml of 1.25M glycine. Cells were harvested and incubated in permeabilization buffer (0.25% triton X-100 in PBS) for 30 min at room temperature. After this, cells were incubated with click reaction buffer (10 mM sodium ascorbate, 2 mM CuSO_4_, 10 μM biotin-azide in PBS). After click reaction, cells were sonicated with lysis buffer (1% SDS in 50 mM Tris–HCl, pH 8.0) and centrifuged for 10 min at 16 100 × g. Protein samples were incubated overnight at 4°C with 50 μl streptavidin–agarose beads. After incubation, beads were washed twice with 1 ml cold lysis buffer for 5 min, once with 1mL of 1 M NaCl, and two additional times with cold lysis buffer. Beads in SDS sample buffer were heated for 25 min at 95°C, analyzed by SDS-PAGE and immunoblotted with indicated antibodies.

### Micronuclei assay

To visualize UV-induced micronuclei, cells were cultured on coverslips and treated with 5 J/m^2^ UV for 48 h. After 3% paraformaldehyde fixation, cells were permeabilized in 0.5% Triton-X solution and stained with DAPI. Micronucleated cells were counted manually from DAPI stained U2OS cells. The micronuclei assays were repeated for three times, and at least 200 cells were counted for each experiment. The Criteria for scoring micronuclei included the following: (i) separate extra-nuclear structures that were rounded shape and DAPI positive and (ii) DAPI staining intensity of cellular micronucleus did not exceed the main nucleus. The DAPI staining intensity and shape of cellular micronuclei were further verified by the visual observations.

### Tissue microarray

The tissue microarrays of ovarian cancer specimen were purchased from US Biomax (# OVC1501). Immunohistochemistry (IHC) was performed as described previously (24). In brief, immunohistochemical staining of USP36 (dilution 1:200), and PrimPol (dilution 1:200) were performed using IHC Select^®^ HRP/DAB kit (Millipore, # DAB150). The images of the sections were independently scored by two pathologists. The extent of staining was scored as 0 (<10%), 1 (11–25%), 2 (26–50%), 3 (51–75%) and 4 (>75%). And the staining intensity was scored as 0 (negative), 1 (weak brown), 2 (moderate brown) and 3 (strong brown). The IHC score was calculated by combining the percentage of positively stained cells with staining intensity. The final staining score >1 was identified as high expression; otherwise, it was identified as low expression. The Pearson correlation coefficient and χ^2^ test were used for statistical analysis of the correlation between USP36 and PrimPol protein levels.

### Statistics

Data in bar and line graphs are presented as mean ± SD of at least three of independent experiments, unless otherwise indicated. The statistical significance of differences between different groups was calculated by two-tailed Student's *t*-test as indicated. A *P*-value <0.05 was considered significant. And statistical significance is represented in all figures by: *P < 0.05; **P < 0.01.

## RESULTS

### Identification of USP36 as a positive regulator of PrimPol

**Figure 1.**
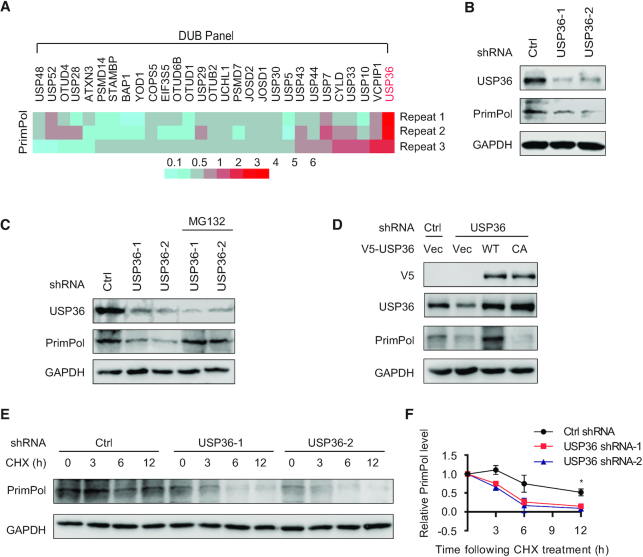
USP36 stabilizes PrimPol protein. (**A**) A panel of deubiquitinases was expressed in HEK293T cells, and the protein levels of PrimPol were assayed using western blot. A heatmap representing the PrimPol changes after overexpression of a panel of deubiquitinases in three repeat experiments. Red and blue represent the increase and decrease in the PrimPol expression level, respectively. With three biological replicates, a total of three deubiquitinases (USP36, USP7 and VCPIP1) were found to upregulate PrimPol protein level (fold-change > 2.0). Among all of them, USP36 was identified to induce the most significant upregulation to PrimPol protein level. (**B**) USP36 depletion reduces PrimPol protein level. HEK293T cells were transduced with lentivirus encoding control (Ctrl) or USP36 shRNAs, and cell lysates were blotted with indicated antibodies. (**C**) HEK293T cells stably expressing Ctrl or USP36 shRNA were treated with vehicle or MG132 (50 μM) for 3 h. Cell lysates were then blotted with indicated antibodies. (**D**) HEK293T cells stably expressing Ctrl or USP36 shRNA-2 were transiently transfected with the wild-type (WT) and catalytically inactive C131A mutant (CA) of V5-tagged USP36. The cells were lysed and western blot was performed with the indicated antibodies. (**E**) HEK293T cells stably expressing Ctrl or USP36 shRNAs were treated with CHX (0.1 mg/ml), and harvested at the indicated times. Cells were lysed and cell lysates were then blotted with the indicated antibodies. (**F**) Quantification of the PrimPol protein levels relative to GAPDH. The graphs represent mean ± SD, two-tailed, Student's *t*-test. *P < 0.05.

DUBs are a large group of proteases that cleave covalently attached ubiquitin from proteins, thus controlling substrate abundance and/or activity (22,25). We overexpressed a panel of Flag-tagged deubiquitinases in HEK293T cells individually, and performed immunoblotting to identify the potential deubiquitinase of PrimPol. Among the proteins in our screening panel, USP36 overexpression induced the most significant upregulation to PrimPol protein level (Figure 1A, and Supplementary Figure S1A–C). We further confirmed the protein levels of PrimPol were dose-dependently upregulated in HEK293T cells following USP36 overexpression (Supplementary Figure S1D). Knock-down of USP36 by two different USP36-specific short hairpin RNAs (shRNAs) reduced PrimPol protein level but not its mRNA level (Figure 1B, and Supplementary Figure S1E), indicating that USP36 regulates PrimPol in a post-transcriptional manner. Moreover, the downregulated PrimPol level after depletion of USP36 was reversed by the addition of MG132, a proteasome inhibitor (Figure 1C), indicating that USP36 regulates PrimPol protein accumulation in a proteasome pathway-dependent manner. Reconstitution of V5-tagged wild-type USP36 (USP36-WT) but not catalytically inactive mutant of USP36 (USP36-CA) in shRNA-2-mediated USP36-depleted cells restored PrimPol protein levels (Figure 1D), suggesting that the USP36 deubiquitination enzyme activity is critical for PrimPol regulation. In addition, we performed cycloheximide (CHX) chase assays to further determine the protein stability of PrimPol. We found that knock-down of USP36 expectedly attenuated the half-life of PrimPol protein in both HEK293T and OVCAR8 cells (Figure 1E, F and Supplementary Figure S2A, B). Conversely, PrimPol stability was significantly increased in HEK293T cells stably expressing Flag-USP36 (Supplementary Figure S1F, G). Further, because PrimPol is important for the nuclear and mitochondrial DNA maintenance (26), we further performed nuclear and mitochondrial fractionations to evaluate whether USP36 has similar effects on PrimPol in the nucleus and mitochondria. Knockdown of USP36 by two different shRNAs reduced PrimPol protein levels in the nucleus and mitochondria, whereas USP36 overexpression upregulated PrimPol expression in both these compartments (Supplementary Figure S1H, I). In addition, given that USP36 is a c-Myc target gene (16), we assessed whether c-Myc plays a role in the stabilization PrimPol. As shown in Supplementary Figure S1J, knockdown of c-Myc did not significantly affect the PrimPol protein level induced by USP36 overexpression. Collectively, these data suggest that USP36 stabilizes PrimPol through its deubiquitinase activity in cells.

### USP36 interacts with PrimPol via its USP domain

**Figure 2.**
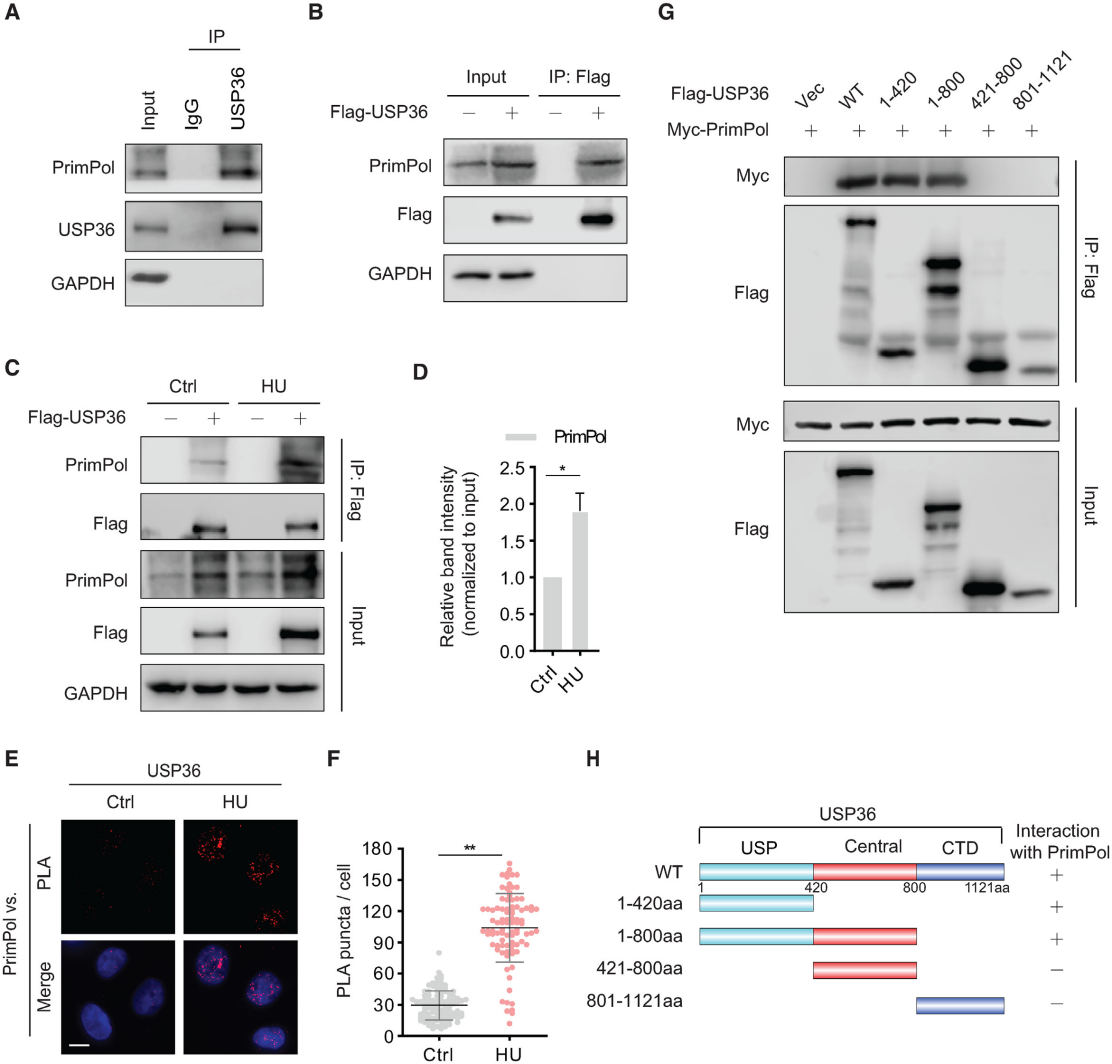
USP36 is a PrimPol binding protein. (**A**) Interaction between endogenous USP36 and PrimPol. HEK293T cell were collected and subjected to immunoprecipitation using control IgG and anti-USP36 antibodies. Blots were then probed with the indicated antibodies. (**B**) Interaction between exogenous USP36 and endogenous PrimPol. Cell lysates from HEK293T cells transiently transfected with Flag-USP36 were subjected to immunoprecipitation with anti-Flag agarose beads, and then blotted with the indicated antibodies. (**C**) HEK293T cells were transiently transfected with Flag-USP36 for 48h, then left untreated or treated with HU (10 mM) for 8 h. The cell lysates were subjected to immunoprecipitation with anti-Flag agarose beads, and immunoprecipitated lysates were equalized for Flag-USP36. Then cell lysates were blotted with the indicated antibodies. (**D**) Quantification of the amount of PrimPol in the pull-down relative to the PrimPol input level. The graphs represent mean ± SD, two-tailed, Student's *t*-test. *P < 0.05. (**E**) In situ PLA between endogenous USP36 and PrimPol after treated with/without HU (10 mM) for 4 h in OVCAR8 cells. Representative images are shown with merged PLA and nuclei (DAPI) channels from PLA experiments. Scale bar in the bottom left is 10 μm. **P < 0.01. Each red dot represents the detection of USP36-PrimPol interaction complex, and the mean ± SD are shown in (**F**). (**G**) The N-terminal USP domain of USP36 is required for binding to PrimPol. HEK293T cells were transiently transfected with Myc-PrimPol together with Wild-type (WT) and truncated mutants (1-420, 1–800, 421–800 and 801–1121aa) of Flag-USP36. The protein interaction was assayed by immunoprecipitation with anti-Flag agarose beads, and then blotted with the indicated antibodies. Schematic representation of the Flag-USP36 and its deletion mutants are shown in (**H**). USP: Ub-specific protease domain. CTD: Carboxy-terminal domain.

In light of the above findings, we tested whether USP36 might physically interact with PrimPol in cells. We detected endogenous binding between USP36 and PrimPol by immunoprecipitation assay (Figure 2A). PrimPol was also detected in immunoprecipitates using anti-Flag agarose beads from cells expressed Flag-USP36 (Figure 2B). Moreover, treatment with the replication-damaging agent hydroxyurea (HU) promoted the interaction between USP36 and PrimPol (Figure 2C, D). We further confirmed the close interaction between USP36 and PrimPol by proximity ligation assay (PLA). Few dots that represent the interaction of USP36 and PrimPol were observed in the nuclei in unstressed cells. Treatment with HU for 4 h and UV for 4 h resulted in a significant increase in the average number of PLA puncta (Figure 2E, F and Supplementary Figure S3A, B).

To identify the region(s) of USP36 mediating PrimPol interactions, we obtained a series of vectors encoding Flag-tagged USP36 deletion mutants. Co-IP analysis displayed that the N-terminal ubiquitin-specific protease (USP) domain of USP36 is both sufficient and necessary for binding PrimPol (Figure 2G, H). Because of PrimPol's role in replication stress (4), we wanted to know whether USP36 can also be recruited to replication forks. We conducted iPOND assays, and found that both USP36 and PrimPol localized to replication forks. And, HU treatment could increase the accumulation of USP36 and PrimPol at replication forks (Supplementary Figure S3C). Given that USP36 can localize to the nucleolus and has an essential role in maintaining nucleolar function (27), we next asked whether USP36 relocated to nucleoplasmic replication forks during replication stress. As expected, we found decreased nucleolus-localized USP36 and increased nucleoplasmic signals after UV treatment (Supplementary Figure S3D). Overall, these results suggest that USP36 interacts with PrimPol at stressed replication forks in cells.

**Figure 3.**
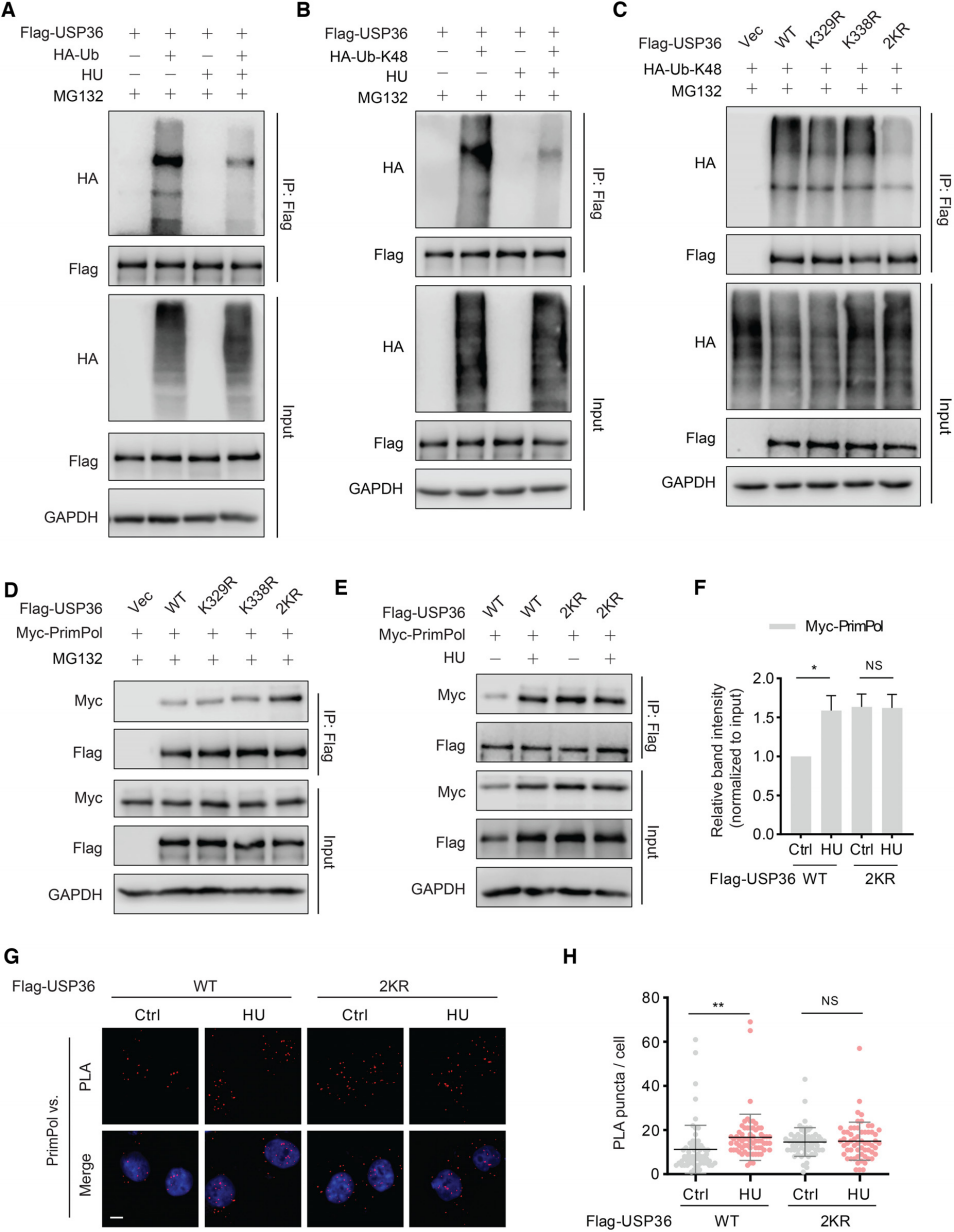
USP36 interacts with PrimPol is dependent on its ubiquitination sites K329 and K338 upon DNA replication stress. (**A**) USP36 stably knocked-down HEK293T cells were transiently transfected with Flag-USP36 and HA-Ub for 48h. Cells were pretreated with HU (10 mM) for 5h, then cotreated with MG-132 (50 μM) for additional 3 h. Cell lysates were subjected to immunoprecipitation with anti-Flag agarose beads, and then blotted with the indicated antibodies. (**B**) USP36 stably knocked-down HEK293T cells were transiently transfected with Flag-USP36 and HA-tagged Ub-K48 for 48h. Cells were pretreated with HU (10 mM) for 5h, then cotreated with MG132 (50 μM) for additional 3 h. Cell lysates were subjected to immunoprecipitation with anti-Flag agarose beads, and then blotted with the indicated antibodies. (**C**) Identification of the ubiquitination sites of USP36 for its K48-specific polyubiquitination. USP36 stably knock-down HEK293T cells were transiently transfected with indicated constructs. After 48 h, cells were treated with MG-132 (50 μM) for 3 h before collecting. Cell lysates were subjected to immunoprecipitation with anti-Flag agarose beads, and then blotted with the indicated antibodies. (**D**) Flag-tagged USP36 expression constructs and Myc-PrimPol plasmid were transfected into USP36 stably knock-down HEK293T cells. After 48 h, cells were treated with MG-132 (50 μM) for 3 h before collecting. Cell lysates were then immunoprecipitated with anti-Flag beads and immunoblotted as indicated. (**E**) USP36 stably knocked-down HEK293T cells were transiently transfected with indicated constructs, then left untreated or treated with HU (10 mM) for 8 h. The cell lysates were subjected to immunoprecipitation with anti-Flag agarose beads, and immunoprecipitated lysates were equalized for Flag-USP36. Then cell lysates blotted with the indicated antibodies. (**F**) Quantification of the amount of Myc-PrimPol in the pull-down relative to the Myc-PrimPol input level. The graphs represent mean ± SD, two-tailed, Student's *t*-test. *P < 0.05; NS = not significant. (**G**) Representative images of merged PLA and nuclei (DAPI) channels from PLA experiments. USP36 stably knock-down OVCAR8 cells were transiently transfected with USP36-wild-type (WT) or the USP36-WT 2KR mutant, then left untreated or treated with HU (10 mM) for 4 h. *In situ* PLA was used to assess the interaction between USP36 and endogenous PrimPol. Scale bar in the bottom left is 10 μm. Each red dot represents the detection of USP36-PrimPol interaction complex, and the graphs represent mean ± SD are shown in (**H**). **P < 0.01; NS = not significant.

Next, we studied how HU treatment promoted the interaction between USP36 and PrimPol. It is now evident that some posttranslational processes, like ubiquitination, have been implicated in the regulation of DUBs (28). We first studied how USP36 ubiquitination is regulated upon replication stress, and found decreased USP36 polyubiquitination following HU treatment (Figure 3A). Given that USP36 protein levels, but not mRNA levels, increased following UV or HU treatment (Supplementary Figure S4A–E), we next examined whether replication stress decreased the K48-specific polyubiquitination of USP36. As shown in Figure 3B, HU treatment significantly down-regulated K48 ubiquitin chain (Ub-K48) on USP36. Moreover, we used Ub-K48 and its mutants, Ub-K48R and found that USP36 polyubiquitination was abrogated by the Ub-K48R mutation (Supplementary Figure S4F). These results could explain the upregulation of USP36 after replication stress. Additionally, HU treatment similarly enhanced the expression of USP36-WT and USP36-CA. PrimPol protein levels in USP36-WT cells, but not USP36-CA cells, increased after HU treatment (Supplementary Figure S4G). These data suggest that increased USP36 by DNA replication stress is not due to its own enzymatic activity. The deubiquitination might be caused by a decrease of E3 ligase activity or activation of another DUB, which remains to be studied in the future. Next, we tried to identify the ubiquitination sites of USP36 that are regulated by replication stress. Recently, two independent groups have reported that the lysine residues, K329 and K338, might be the potential ubiquitination sites of USP36 regulated by DNA damage (29,30). Accordingly, we generated USP36-WT K329R and K338R mutants, and found that single site mutants (K329R and K338R) had little effect on the K48-linked polyubiquitination, while the two K to R mutants (2KR) almost abolished the K48-linked polyubiquitination on USP36 (Figure 3C). Next, we tested whether these two lysine residues (K329R and K338R) were essential for the regulation of USP36-PrimPol interaction. We found that the 2KR mutation enhanced their interaction even in unstressed cells (Figure 3D). However, in contrast to USP36-WT, treatment with HU did not further enhance the interaction between USP36-WT-2KR and PrimPol (Figure 3E, F). The PLA assay also confirmed the increased interaction of USP36-WT-2KR and PrimPol in the nuclei in unstressed cells, which could not be further enhanced by HU treatment (Figure 3G, H). Together, these findings clearly establish the essential roles of K329 and K338 of USP36, whose deubiquitination is important for induced interaction with PrimPol upon DNA replication stress.

### USP36 deubiquitinates PrimPol, especially the K29-linked polyubiquitin chains

**Figure 4.**
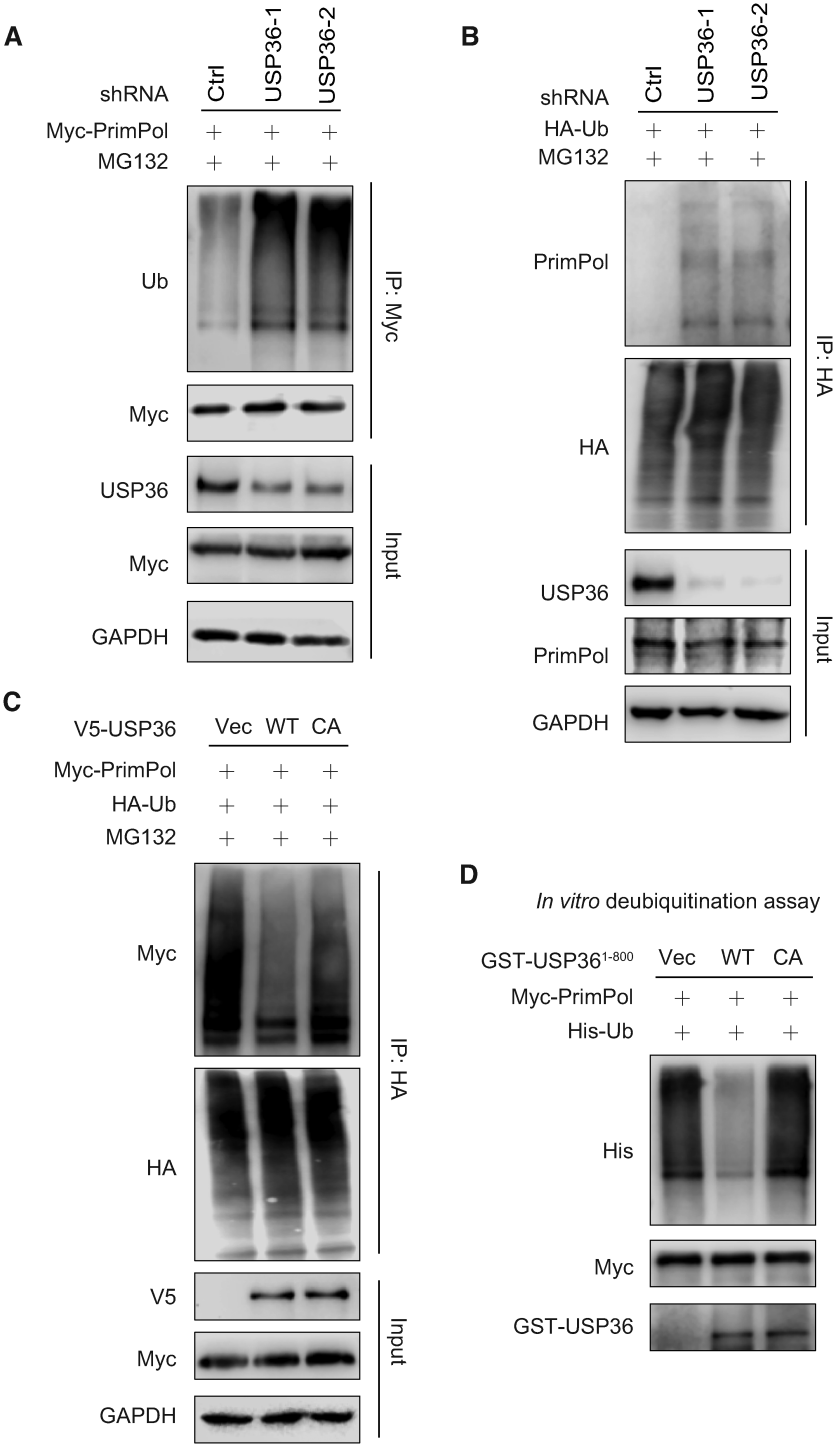
USP36 deubiquitinates PrimPol. (**A**) HEK293T cells stably expressing control (Ctrl) or USP36 shRNAs were transiently transfected with Myc-tagged PrimPol. After 48 h, cells were treated with MG132 (50 μM) for 3 h. Cell lysates were subjected to immunoprecipitation with anti-Myc agarose beads, and then blotted with the indicated antibodies. (**B**) HEK293T cells stably expressing Ctrl or USP36 shRNAs were transiently transfected with HA-tagged ubiquitin. After 48 h, cells were treated with MG132 (50 μM) for 3 h. Cell lysates were subjected to immunoprecipitation with anti-HA agarose beads, and then blotted with the indicated antibodies. (**C**) HEK293T cells with USP36 stably knocked-down were transiently transfected with indicated constructs. After 48 h, cells were treated with MG132 (50 μM) for 3 h before collecting. Cell lysates were subjected to immunoprecipitation with anti-HA agarose beads, and then blotted with the indicated antibodies. (**D**) Deubiquitination of PrimPol *in vitro* by USP36. Ubiquitinated PrimPol was incubated with purified USP36^1–800^-WT or USP36^1–800^-CA *in vitro*. Cell lysates were subjected to immunoprecipitation with anti-Myc agarose beads, and then blotted with the indicated antibodies.

Because we found that USP36 stabilizes PrimPol through its deubiquitinase activity in cells, we next evaluated the roles of USP36 on PrimPol ubiquitination. We found that USP36 knock-down in both HEK293T and OVCAR8 cells significantly increased the polyubiquitination of PrimPol compared to control (Ctrl) cells (Figure 4A, and Supplementary Figure S5A). Under denaturing conditions, IP of ubiquitinated proteins followed by immunoblot assay of PrimPol also showed increased polyubiquitination of endogenous PrimPol in USP36 knock-down cells (Figure 4B). In addition, in USP36-depleted cells, overexpression of USP36-WT, but not USP36-CA mutant, resulted in a reduction in PrimPol polyubiquitination (Figure 4C, and Supplementary Figure S5B). To determine whether USP36 directly deubiquitinates PrimPol *in vitro*, we purified ubiquitinated PrimPol from HEK293T cells co-transfected with Myc-PrimPol and His-Ub using affinity purification. The polyubiquitinated PrimPol was incubated with purified recombinant USP domain-containing GST-USP36^1–800^ (WT or CA) or control buffer followed by immunoblot with anti-Ub antibody. USP36^1–800^-WT also interacted with PrimPol (Figure 2G-H). As shown in Figure 4D, USP36^1–800^-WT, but not USP36^1–800^-CA mutant, reduced the PrimPol polyubiquitination. Taken together, these results suggest that USP36 could be a bona fide deubiquitinase targeting PrimPol protein for deubiquitination.

**Figure 5.**
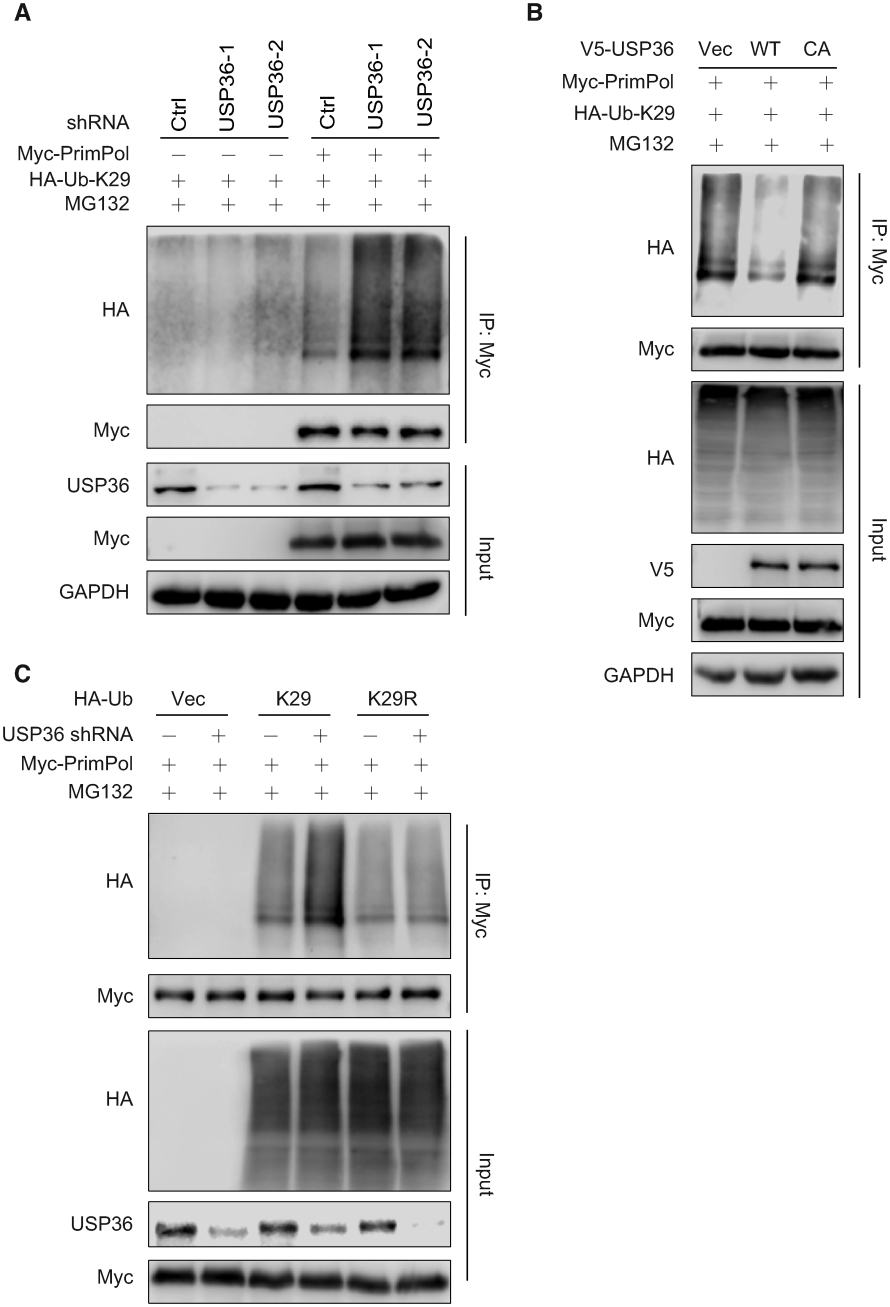
USP36 regulates K29-linked polyubiquitination of PrimPol. (**A**) Knock-down of USP36 promotes K29-linked polyubiquitination of PrimPol. HEK293T cells stably expressing control (Ctrl) or USP36 shRNAs were transiently transfected with HA-tagged Ub-K29 and Myc-tagged PrimPol. After 48 h, cells were treated with MG132 (50 μM) for 3 h before collecting. Cell lysates were subjected to immunoprecipitation with anti-Myc agarose beads, and then blotted with the indicated antibodies. (**B**) USP36 stably knock-down HEK293T cells were transiently transfected with indicated constructs. After 48 h, cells were treated with MG132 (50 μM) for 3 h before collecting. Cell lysates were subjected to immunoprecipitation with anti-Myc agarose beads, and then blotted with the indicated antibodies. (**C**) Ctrl or USP36 stably knock-down HEK293T cells were transiently transfected with indicated HA-K29 lysine-specific mutant constructs and Myc-tagged PrimPol. After 48 h, cells were treated with MG132 (50 μM) for 3 h before collecting. Cell lysates were subjected to immunoprecipitation with anti-Myc agarose beads, and then blotted with the indicated antibodies.

It is well known that ubiquitin has several lysine residues (Ub-K6, Ub-K11, Ub-K27, Ub-K29, Ub-K33, Ub-K48 and Ub-K63), which can be used to form distinct linkage types of ubiquitin chains and perform different cellular functions (31). To further understand the features of USP36-mediated PrimPol deubiquitination, we set out to determine the types of ubiquitin linkage using a series of ubiquitin mutant plasmids in which only one lysine was retained. USP36-depleted cells were transfected with one of these ubiquitin mutants and Myc-PrimPol, followed by ubiquitination assays. Interestingly, we found that PrimPol was extensively ubiquitinated with Ub-K11, Ub-K27 and Ub-K29 linkages (Supplementary Figure S6A). We further examined these linkages (Ub-K11, Ub-K27 or Ub-K29) with or without USP36 knock-down, and found that USP36 depletion only increased K29-linked polyubiquitination of PrimPol (Figure 5A, and Supplementary Figure S6B). Furthermore, in USP36-depleted cells, expression of USP36-WT but not USP36-CA mutant resulted in a reduction in PrimPol K29-linked polyubiquitination (Figure 5B). Using Ub-K29 and its mutant Ub-K29R, we found that the USP36 depletion induced increase in polyubiquitination was abrogated by the Ub-K29R mutation (Figure 5C). However, we still observed polyubiquitin chains of PrimPol in cell expressing K29R, suggesting that other types of ubiquitin chains might be not affected by USP36. In addition, given that the *Drosophila* homolog of USP36 (dUSP36) has been reported to hydrolyze K63-polyUb chains (32), we wanted to know whether USP36 affected the K63-specific ubiquitination of PrimPol. As shown in Supplementary Figure S6C, no significant change of PrimPol K63-linked polyubiquitination could be induced by USP36 depletion. Our data unambiguously show that USP36 deubiquitinates PrimPol through K29-linked polyubiquitin chains.

### USP36 participates in DNA replication stress through a PrimPol-dependent manner

As PrimPol is reported to play a crucial role in DNA replication stress (15,33), we first examined whether the expression of USP36 is influenced by treatment with different types of replication stress, including UV and HU. PrimPol expression levels increased following UV or HU treatment (Supplementary Figure S4A–D), as has been reported previously (34). Intriguingly, the USP36 protein levels, but not mRNA levels, were similar and followed the same trend (Supplementary Figure S4A–E). Furthermore, knock-down of USP36 attenuated replication stress-induced PrimPol upregulation (Supplementary Figure S4A–D).

**Figure 6.**
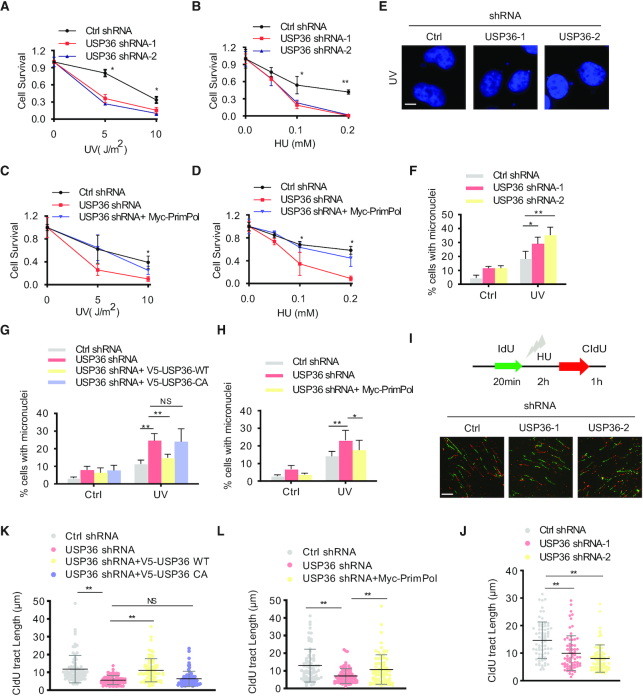
USP36 participates in DNA replication stress through a PrimPol-dependent manner. (**A, B**) OVCAR8 cells from Supplementary Figure S7A were treated with indicated does of UV or HU. Cell survival was determined by colony formation assay. Error bars represent ± SD from three independent experiments. (**C, D**) OVCAR8 cells from Supplementary Figure S7B were treated with indicated does of UV or HU. Cell survival was determined by colony formation assay. Error bars represent ± SD from three independent experiments. (**E**) Representative images of micronuclei labeled with the DNA dye DAPI in OVCAR8 USP36-knockdown cells from Supplementary Figure S7A. Cells were left untreated or treated with UV (5 J*/*m^2^) for 48 h. Cells were fixed, permeabilized in 0.5% Triton X-100, stained with DAPI, then analyzed by Nikon eclipse 80i fluorescence microscope for micronuclei. The micronuclei assays were repeated for three times, and at least 200 cells were counted for each experiment. Scale bar in the bottom left is 10 μm. (**F**) Quantification of micronuclei in cells from E. (**G**) OVCAR8 USP36-knockdown cells reconstituted with V5-tagged USP36 (WT and C131A mutant) from Supplementary Figure S7C were used for measuring micronuclei. (**H**) OVCAR8 USP36-knockdown cells reconstituted with Myc-PrimPol from Supplementary Figure S7B were used for measuring micronuclei. (**I**) Representative images of DNA fibers in OVCAR8 USP36-knockdown cells from Supplementary Figure S7A. Cells were labeled with IdU for 20 minutes as a control to label the active replication, and followed by incubation with 4 mM HU for 2 h. Cells were then labeled with CIdU for 1h to evaluate the DNA replication upon genotoxic stress. DNA fibers were stretched on a microscope slide, stained with IdU and CIdU antibodies, imaged, and the lengths of fiber tracks measured. Scale bar in the bottom left is 10 μm. (**J**) Quantification of DNA fibers in cells from I. (**K**) OVCAR8 USP36-knockdown cells reconstituted with V5-tagged USP36 (WT and C131A mutant) from Supplementary Figure S7C were used to detect the restart efficiency of stalled replication forks. (**L**) OVCAR8 USP36-knockdown cells reconstituted with Myc-PrimPol from Supplementary Figure S7B were used to detect the restart efficiency of stalled replication forks. The graphs represent mean ± SD, two-tailed, Student's *t*-test. *P < 0.05; **P < 0.01; NS = not significant.

Next, we evaluated whether USP36-depleted cells showed an increased sensitivity to these DNA replication stress-inducing agents. We found that knock-down of USP36 caused hypersensitivity to UV and HU in a human ovarian cancer cell line (OVCAR8) (Figure 6A-B, and Supplementary Figure S7A). Notably, restoring PrimPol expression in USP36-depleted cells reversed the hypersensitivity to UV and HU (Figure 6C, D and Supplementary Figure S7B). These results suggest that USP36 regulates the cellular response to DNA-damaging agents in a PrimPol-dependent manner.

Previous studies showed that another indication of replication stress is a higher percentage of interphase cells with micronuclei after downregulation of PrimPol (26). We also observed an increased frequency of micronuclei in USP36 knock-down OVCAR8 cells (Figure 6E, F and protein expression is shown in Supplementary Figure S7A). Reconstitution of V5-tagged USP36-WT but not USP36-CA in USP36-depleted (shRNA-2) OVCAR8 cells reversed the increase of micronuclei (Figure 6G, and Supplementary Figure S7C). Meanwhile, ectopic expression of PrimPol in USP36-depleted OVCAR8 and PEO1 cells completely reversed the increase of micronuclei (Figure 6H, Supplementary Figure S7D and protein expression is shown in Supplementary Figure S7B). These findings indicate that USP36 regulates genomic stability through PrimPol.

To better characterize whether USP36-deficient cells have a defective response upon replication stress, we performed DNA fiber assays. Quantification of CIdU track length shows that under replication stress conditions, USP36-depleted OVCAR8 cells exhibited a significant decrease in replication strand lengths compared to Ctrl cells (Figure 6I-J, and protein expression is shown in Supplementary Figure S7A). Reconstitution of V5-tagged USP36-WT but not USP36-CA in USP36-depleted OVCAR8 cells partially restored replication strand lengths (Figure 6K, and protein expression is shown in Supplementary Figure S7C). Additionally, the decreased replication strand lengths induced by USP36 knock-down were efficiently reversed by the ectopic expression of PrimPol in both OVCAR8 and PEO1 cells (Figure 6L, Supplementary Figure S7E, and protein expression is shown in Supplementary Figure S7B). These results reveal that USP36 regulates the restart efficiency of stalled replication forks in a PrimPol-dependent manner.

In addition, we knocked down USP36 in OVCAR8 cells using two different USP36-specific shRNAs and assessed γH2AX foci, a hallmark of replication fork stalling (35). USP36 status had little effect on the initial induction of γH2AX induced by HU, whereas γH2AX signals remained higher in USP36 knock-down cells than Ctrl cells over the 24 h time course after HU release (Supplementary Figure S7F, G and protein expression is shown in Supplementary Figure S7A). Taken together, the above-mentioned findings demonstrate that USP36 plays an important role in replication stress response through regulating PrimPol.

### USP36 expression is correlated with the level of PrimPol in ovarian cancer and regulates the treatment response

**Figure 7.**
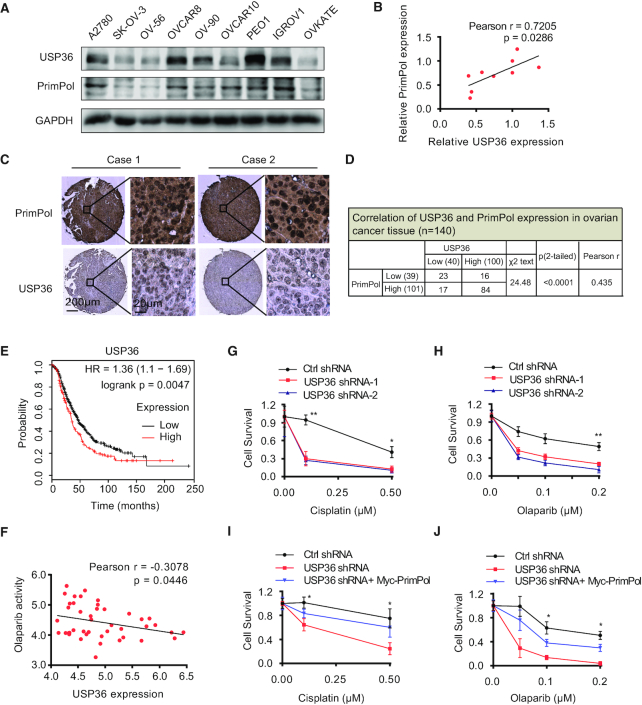
USP36 expression is correlated with PrimPol levels in ovarian cancer, and regulates the treatment response of cancer cells through PrimPol stabilization. (**A**) Cell lysates from several ovarian cancer cell lines were blotted with USP36 and PrimPol antibodies. (**B**) Correlation analysis of USP36 and PrimPol in ovarian cancer cells. Statistical analyses were performed with the *χ*^2^ test. The Pearson *r* indicates correlation coefficient. (**C**) Representative images of immunohistochemical staining of USP36 and PrimPol on tissue microarray of ovarian cancer specimens (*n* = 140). Scale bars are indicated. (**D**) USP36 expression correlates with PrimPol levels in tissue microarray of ovarian cancer samples. Protein levels of USP36 and PrimPol were quantified in ovarian cancer specimens. Statistical analyses were performed with the *χ*^2^ test. (**E**) Survival analysis of ovarian cancer patients by the Kaplan–Meier Plotter. (**F**) Negative correlation between olaparib activity and USP36 expression in 43 ovarian cancer cells using CellMinerCDB. (**G-H**) Control and OVCAR8 cells with USP36 stably knock-down from Supplementary Figure S7A were subjected to cisplatin or olaparib treatment. Survival of the cells was assessed by colony formation assay. (**I, J**) OVCAR8 USP36-knockdown cells reconstituted with Myc-PrimPol from Supplementary Figure S7B were subjected to cisplatin or olaparib treatment. Survival of the cells was assessed by colony formation assay. Error bars represent ± SD from three independent experiments. *P < 0.05; **P < 0.01.

Recent findings have reported that the upregulation and increased chromatin recruitment of the PrimPol protein in ovarian cancer cells (34). Until now, our hypothesis is that USP36 upregulation causes PrimPol protein deubiquitination and stabilization, which might affect chemotherapy response. To test our hypothesis, we examined the correlation between the expression of USP36 and PrimPol proteins in human ovarian cancer cells and specimens. As shown in Figure 7A-B, a positive correlation between USP36 and PrimPol protein levels (*P* = 0.0286, Pearson *r* = 0.7205) was observed in ovarian cancer cell lines. We then performed immunohistochemical staining of USP36 and PrimPol in an ovarian cancer tissue microarray containing a cohort of ovarian cancer samples (n = 140). Representative images of staining of USP36 and PrimPol are shown in Figure 7C. The majority of tumors expressed a high level of USP36 and PrimPol protein, and we observed a significantly positive correlation between USP36 and PrimPol (*P* < 0.0001, Pearson *r* = 0.435) as 84% (84 of 100) of USP36-high samples had high level of PrimPol (Figure 7D). These data indicate that upregulated expression of USP36 correlates with increased expression of PrimPol in ovarian cancer.

In addition, given PrimPol-mediated adaptive response to multiple drugs, such as cisplatin, we evaluated whether USP36 plays a role in response to therapy in ovarian cancer cells. The Kaplan–Meier Plotter tool (36) was used to evaluate the correlation of USP36 expression with clinical outcome in ovarian cancer patients. The clinical data suggest that the upregulation of USP36 expression was significantly related to shorter overall survival (Figure 7E). Meanwhile, USP36 expression was negatively associated with the sensitivity of cancer cells to the anti-cancer drug olaparib, as evidenced by analysis of the data from the CellMinerCDB (37) in 43 ovarian cancer cell lines (Figure 7F). These findings suggest that high USP36 expression is correlated with poor therapeutic response and poor prognosis in ovarian cancer patients.

Finally, to establish a causal effect for USP36 in chemosensitivity, we sought to evaluate whether targeting USP36 could sensitize ovarian cancer cells to cisplatin and olaparib. Indeed, the cytotoxicity of cisplatin or olaparib against ovarian cancer cells OVCAR8 and PEO1 was increased after knock-down of USP36, as compared to Ctrl cells (Figure 7G, H, protein expression is shown in Supplementary Figure S7A, and Supplementary Figure S8A–C). Furthermore, ectopic expression of PrimPol in USP36-depleted OVCAR8 and PEO1 cells completely reversed the cytotoxicity of cisplatin or olaparib (Figure 7I, J, Supplementary Figure S8D-E and protein expression is shown in Supplementary Figure S7B). Taken together, these data support an important role of USP36 in regulating the response of cancer cells to anti-cancer drugs through PrimPol stabilization.

## DISCUSSION

PrimPol is an important component to maintain adaptive DNA replication by repriming replication restart downstream of replication stalling lesions and structures (38,39). Understanding how cells adapt to genotoxic agents and how PrimPol mediates responses in replication fork restart has become increasingly important to optimize tumor therapy. To date, few studies focus on the detailed mechanisms of PrimPol regulation after exposure to multiple replication stalling agents. Previous studies showed that PrimPol's interactor RPA is critical for the recruitment of PrimPol to the stalled replication fork, contributing to replication restart (11). Deletion of the C-terminal RPA-binding motif of PrimPol impairs its interaction with RPA, and thus weakens the primase and polymerase activities of PrimPol (40,41). However, whether and how PrimPol itself is regulated following DNA replicative stress is still unclear. Thus, the identification and characterization of the underlying signaling pathway modulating PrimPol stabilization will be of importance to establish its biological function, and can be exploited for promising therapeutic interventions.

**Figure 8.**
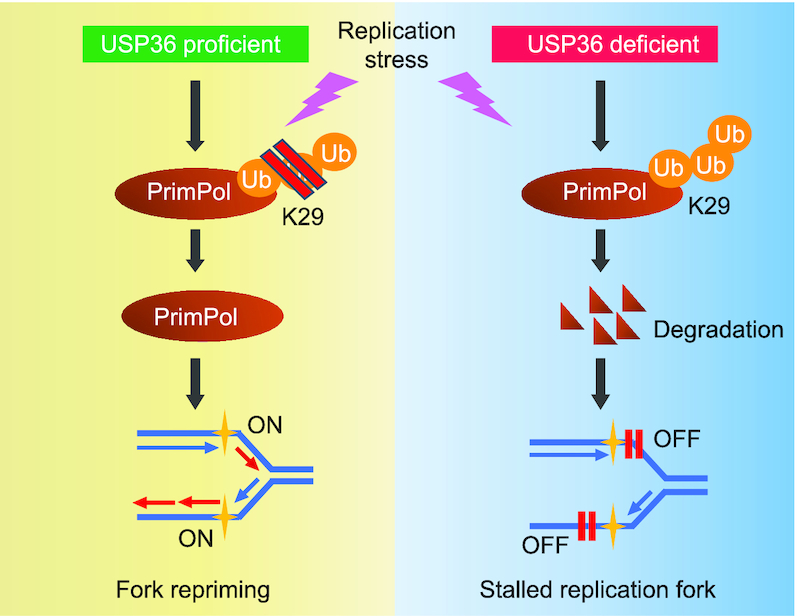
A schematic for PrimPol stabilization by deubiquitinase USP36, which promotes replication fork progression following DNA replication stress. Overexpressed USP36 binds to PrimPol and interferes with its K29-linked polyubiquitination and degradation. The PrimPol protein is markedly stabilized by USP36 overexpression and promotes stalled fork restart in the response to DNA replication stress in cancer cells.

The ubiquitin-specific protease, USP36, has been reported to interact with histone 2b (H2B) and deubiquitinates the monoubiquitinated H2B *in vitro* (42). Moreover, USP36 has been shown to be overexpressed in multiple types of human cancers, and might have oncogenic activity. Overexpression of USP36 promotes cancer cell proliferation through deubiquitinating and stabilizing c-Myc protein in the nucleolus (43). More recently, it has been shown that USP36 enhances CHD7 protein stability by direct deubiquitination, subsequently facilitating tumor progression (44). Here, our studies indicate that USP36 is crucial in maintaining stalled fork restart following replication stress (Figure 8). Mechanistically, USP36 deubiquitinates and stabilizes PrimPol. Cancer cells generally have higher replication stress, and the USP36-PrimPol axis might be required to adapt to this stress. Indeed, we also demonstrate upregulated USP36 in ovarian cancer patients that correlates with higher PrimPol level and poor prognosis. Our results also suggest targeting USP36 might be effective to sensitize cells to chemotherapy. As a proof of concept for targeting this pathway, we show that depletion of USP36 sensitizes ovarian cancer cells to therapeutic response by inhibiting PrimPol-mediated fork restart. Our study reveals a novel mechanism governing PrimPol protein stability by USP36 in human cancers.

The dynamic and highly conserved ubiquitination modification of proteins is a tightly controlled biological process, which has emerged as an important mechanism for several pathologies (45,46). Seven lysine residues on ubiquitin can be modified for the synthesis of polyubiquitin chains on protein substrates, contributing the maintaining protein homeostasis (47). Especially, all non-K63 linkages may target proteins for degradation, supporting the notion that non-K63 polyubiquitin chains are critical for the ubiquitin-proteasome system function (48). Among these, K29 polyubiquitination plays an important role in proteasome-mediated protein degradation, involving in DNA damage response (49). Depletion of the ubiquitin ligase HUWE1 reduces the K29-linked polyubiquitination of JMJD1A, thereby impairing DNA damage repair and sensitizing prostate cells to topoisomerase inhibitors or the PARP inhibitor olaparib (50). The ubiquitin-binding Ufd1-Npl4 complex were shown to be required for disassembly of ubiquitylated CMG helicase during DNA replication termination in eukaryotes, followed by K29-dependent degradation of Mcm7 (51). In our study, USP36 exhibits a strong preference for K29-linked ubiquitin peptides on PrimPol. Based on the findings here, we would suggest that deubiquitinase USP36 attenuates the K29-linked polyubiquitination of PrimPol, which could induce PrimPol stability contributing to DNA replication.

In conclusion, our work introduces the important concept that a deubiquitinase cooperates with the dual primase–polymerase PrimPol on replication repriming in replication stress. We envision that USP36 is important for genome stability. On the other hand, in cancer cells, higher expression of USP36 might render cancer cells resistant to replication stress, while lower USP36 level or inhibition of USP36-PrimPol axis might sensitize cancer cells to genotoxic insults. In future work, it will be important to screen and identify USP36 specific inhibitors that suppress USP36 activity in cancer cells and that will consequently inhibit PrimPol-dependent repriming and sensitize ovarian cancer to therapeutic drugs.

## DATA AVAILABILITY

All correspondence and material requests should be addressed to ZL.

## Supplementary Material

gkaa1090_Supplemental_File
