# Supplementary material for: The deubiquitinase USP36 Regulates DNA replication stress and confers therapeutic resistance through PrimPol stabilization
Source: Nucleic Acids Res. 2020 Nov 25;48(22):12711–26. doi: 10.1093/nar/gkaa1090 (PMC7736794; doi:10.1093/nar/gkaa1090)
Supplement: gkaa1090_Supplemental_File [file gkaa1090_supplemental_file.pdf]

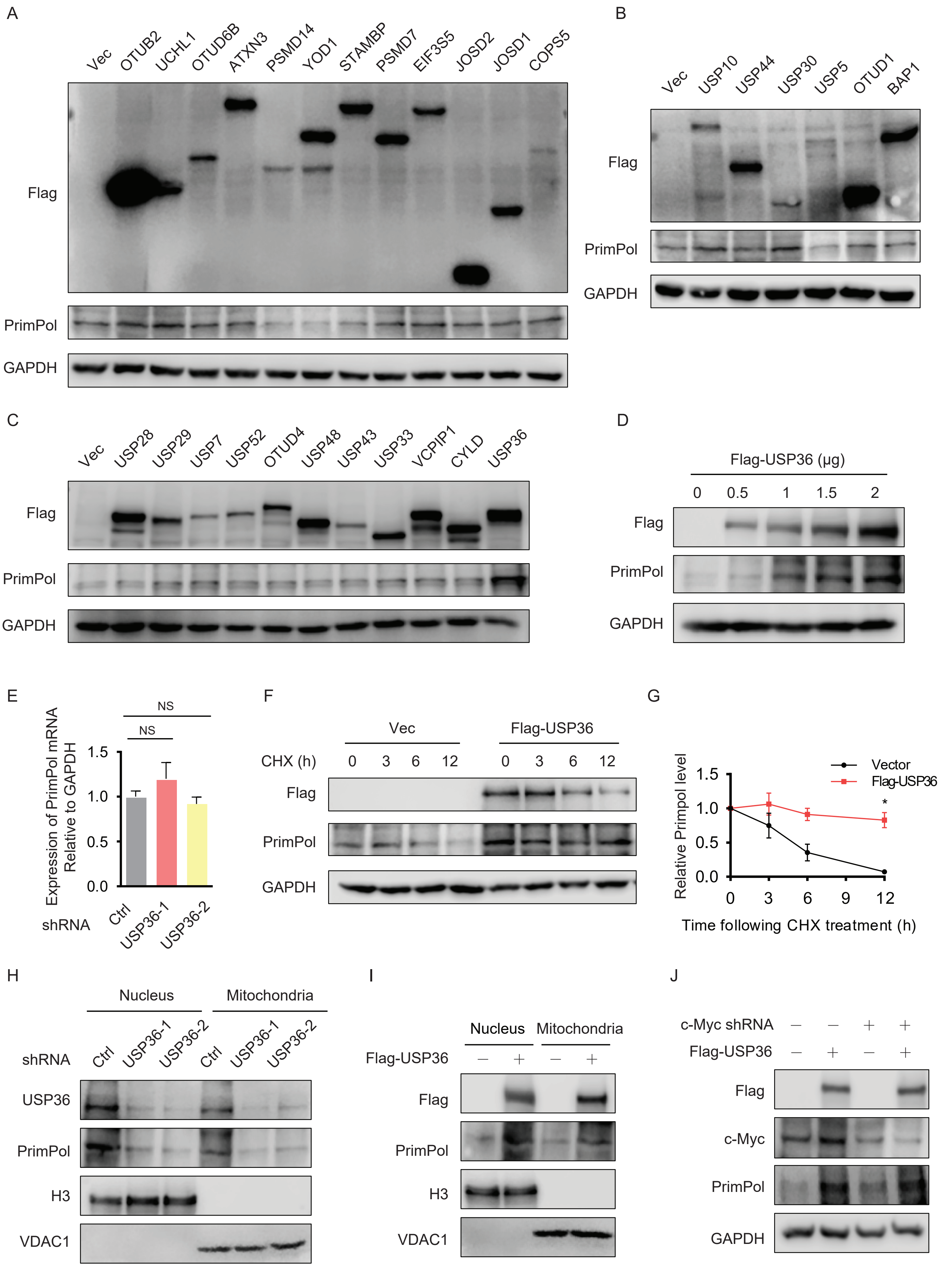
A
Vec
OTUB2
UCHL1
OTUD6B
ATXN3
PSMD14
YOD1
STAMBP
PSMD7
EIF3S5
JOSD2
JOSD1
COPS5
Flag
PrimPol
GAPDH
B
Vec
USP10
USP44
USP30
USP5
OTUD1
BAP1
Flag
PrimPol
GAPDH
C
Vec
USP28
USP29
USP7
USP52
OTUD4
USP48
USP43
USP33
VCPIP1
CYLD
USP36
Flag
PrimPol
GAPDH
D
Flag-USP36 (μg)
0
0.5
1
1.5
2
Flag
PrimPol
GAPDH
E
Expression of PrimPol mRNA
Relative to GAPDH
NS
NS
1.5
1.0
0.5
0.0
shRNA
Ctrl
USP36-1
USP36-2
F
Vec
Flag-USP36
CHX (h)
0
3
6
12
0
3
6
12
Flag
PrimPol
GAPDH
G
Relative Primpol level
Vector
Flag-USP36
*
1.5
1.0
0.5
0.0
0
3
6
9
12
Time following CHX treatment (h)
H
Nucleus
Mitochondria
shRNA
Ctrl
USP36-1
USP36-2
Ctrl
USP36-1
USP36-2
USP36
PrimPol
H3
VDAC1
I
Nucleus
Mitochondria
Flag-USP36
–
+
–
+
Flag
PrimPol
H3
VDAC1
J
c-Myc shRNA
–
–
+
+
Flag-USP36
–
+
–
+
Flag
c-Myc
PrimPol
GAPDH

Supplementary Figure S1. USP36 stabilizes PrimPol protein.

(A-C) HEK293T cells were transiently transfected with a panel of deubiquitinases. The protein levels of PrimPol and Flag-tagged deubiquitinases were detected by western blot with indicated antibodies. (D) Gradient amounts of Flag-USP36 plasmids transiently transfected in HEK293T cells, and the expression of PrimPol protein was detected by western blot. (E) mRNAs were extracted from control or USP36 shRNAs stably expressing HEK293T cells, and subjected to qRT-PCR with the PrimPol primers. The data shown are mean ± SD of three independent experiments. Statistical analyses were performed with the two-tailed Student's t-test. NS = not significant. (F) HEK293T cells were transiently transfected with vector or Flag-USP36 and treated with CHX (0.1 mg/ml). The cell lysates were harvested at the indicated time points, then blotted with the indicated antibodies. (G) Quantification of the PrimPol protein levels relative to GAPDH. The graphs represent mean ± SD, two-tailed, Student's t-test. *$p < 0.05$. (H-I) Isolated nuclear and mitochondrial extracts from HEK293T cells after USP36 knock-down (H) or overexpression (I) were blotted with the indicated antibodies. H3 and VDAC1 were used as a loading control for the nuclear and mitochondrial extracts, respectively. (J) HEK293T cells expressing Ctrl or c-Myc shRNA were transiently transfected with the Flag-USP36. Cell lysates were then blotted with the indicated antibodies.

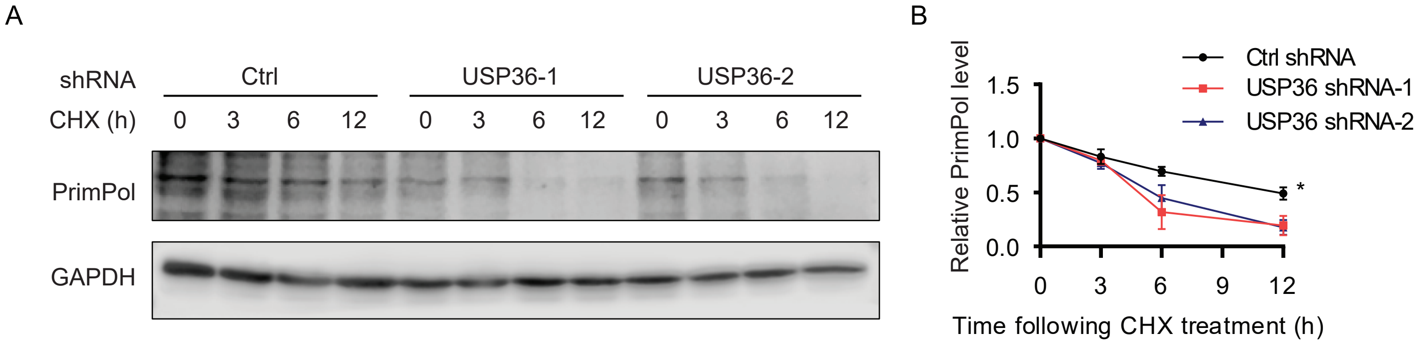
A
shRNA
Ctrl
USP36-1
USP36-2
CHX (h)
0 3 6 12 0 3 6 12 0 3 6 12
PrimPol
GAPDH
B
Ctrl shRNA
USP36 shRNA-1
USP36 shRNA-2
Relative PrimPol level
1.5
1.0
0.5
0.0
*
0 3 6 9 12
Time following CHX treatment (h)

Supplementary Figure S2. USP36 knock-down attenuates the half-life of PrimPol protein.

(A) OVCAR8 cells stably expressing Ctrl or USP36 shRNAs were treated with CHX (0.1 mg/ml), and harvested at the indicated times. Cells were lysed and cell lysates were then blotted with the indicated antibodies. (B) Quantification of the PrimPol protein levels relative to GAPDH. The graphs represent mean ± SD, two-tailed, Student's t-test. *$p < 0.05$.

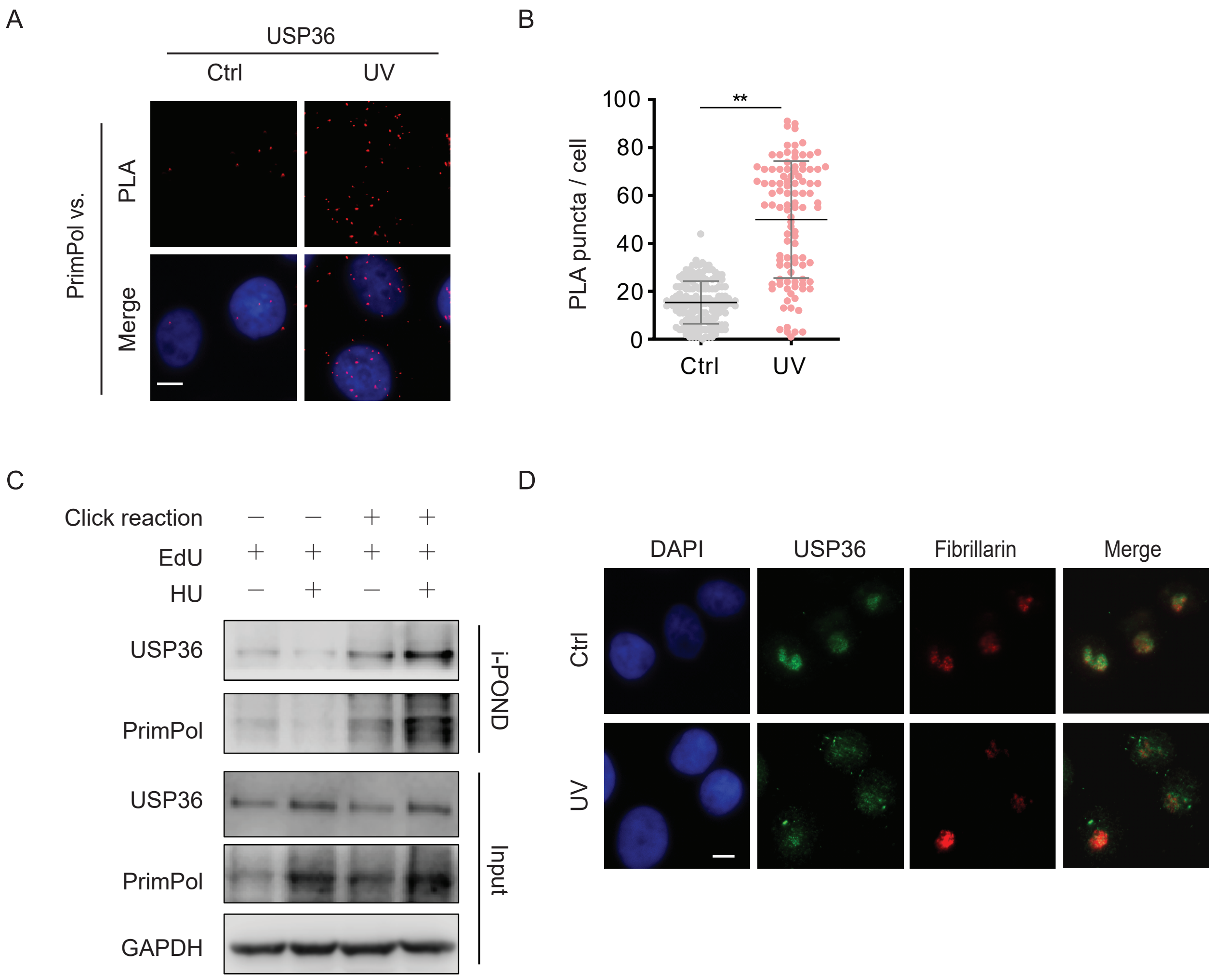
A
USP36
Ctrl
UV
PrimPol vs.
PLA
Merge
B
PLA puncta / cell
100
80
60
40
20
0
**
Ctrl
UV
C
Click reaction
EdU
HU
USP36
PrimPol
USP36
PrimPol
GAPDH
i-POND
Input
D
DAPI
USP36
Fibrillarin
Merge
Ctrl
UV

Supplementary Figure S3. USP36 interacts with PrimPol at replication forks.

(A) In situ PLA between endogenous USP36 and PrimPol after treated with/without UV (5 J/m^2) for 4 h in OVCAR8 cells. Representative images are shown with merged PLA and nuclei (DAPI) channels from PLA experiments. Scale bar in the bottom left is 10 μm. Each red dot represents the detection of USP36-PrimPol interaction complex, and the mean ± SD are shown in (B). **p < 0.01. (C) HEK293T cells were incubated with EdU 10 μM for 10 min and HU 10 mM for 4 h. Replication fork proteins were isolated by iPOND and immunoblotted with indicated antibodies. (D) Double immunofluorescence to detect USP36 (green) and the nucleolar marker Fibrillarin (red) in OVCAR8 cells treated with/without UV (5 J/m^2) for 8 h. Scale bar in the bottom left is 10 μm.

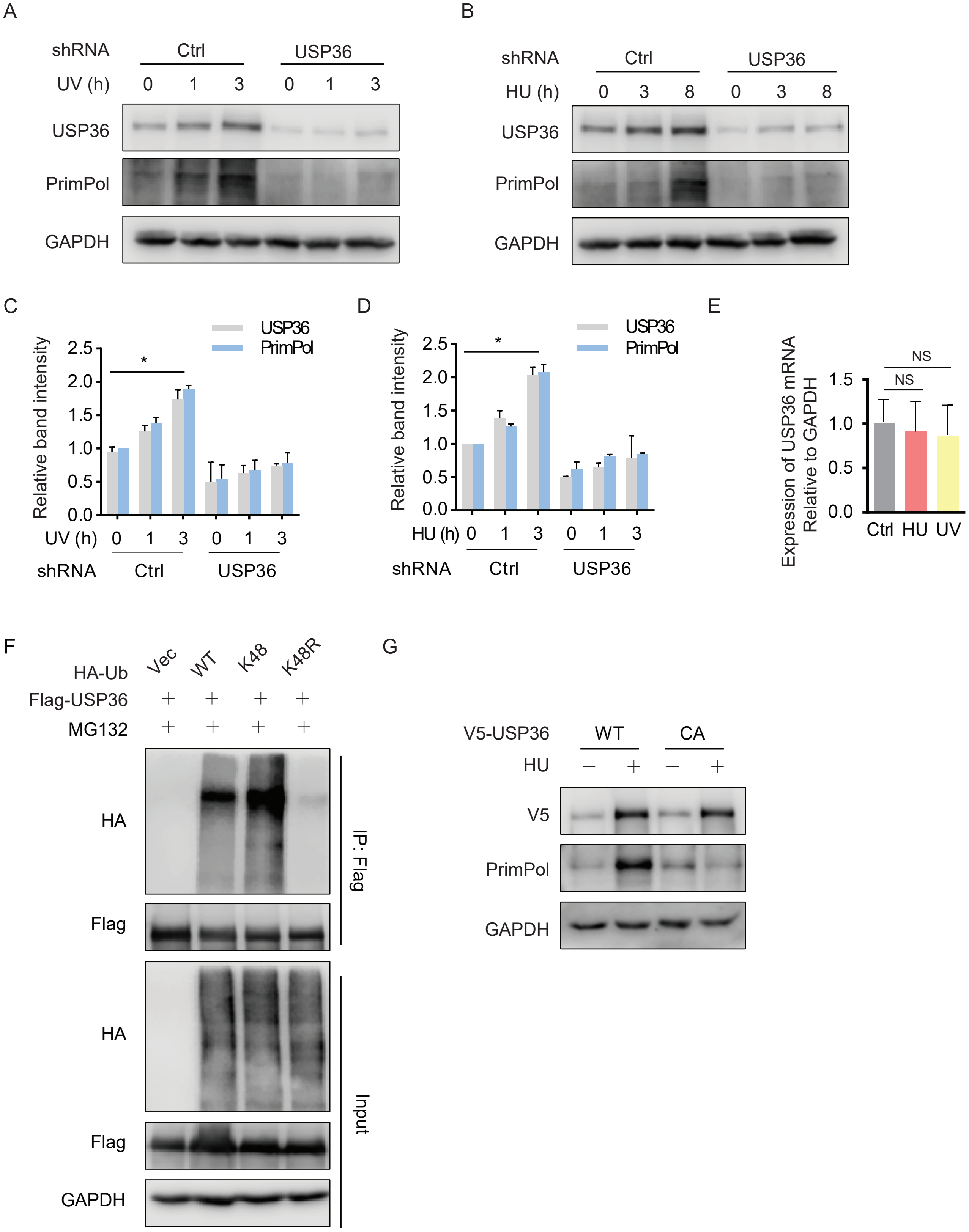

A
shRNA
Ctrl
USP36
UV (h)
0 1 3 0 1 3
USP36
PrimPol
GAPDH
B
shRNA
Ctrl
USP36
HU (h)
0 3 8 0 3 8
USP36
PrimPol
GAPDH
C
USP36
PrimPol
Relative band intensity
2.5
2.0
1.5
1.0
0.5
0.0
*
UV (h) 0 1 3 0 1 3
shRNA Ctrl USP36
D
USP36
PrimPol
Relative band intensity
2.5
2.0
1.5
1.0
0.5
0.0
*
HU (h) 0 1 3 0 1 3
shRNA Ctrl USP36
E
Expression of USP36 mRNA
Relative to GAPDH
1.5
1.0
0.5
0.0
NS
NS
Ctrl HU UV
F
HA-Ub
Vec WT K48 K48R
Flag-USP36
+ + + +
MG132
+ + + +
HA
Flag
IP: Flag
HA
Flag
GAPDH
Input
G
V5-USP36
WT
CA
HU
– + – +
V5
PrimPol
GAPDH

Supplementary Figure S4. The replication stress affects K48 ubiquitin chain on USP36.

(A-B) HEK293T cells stably expressing control (Ctrl) or USP36 shRNAs were left untreated or treated with UV (5 J/m2), or HU (10 mM) and harvested at the indicated times. Cells lysates were then blotted with the indicated antibodies. (C-D) Quantification of the USP36 and PrimPol protein levels relative to GAPDH. The graphs represent mean ± SD, two-tailed, Student's t-test. *p < 0.05. (E) After treatment with UV (5 J/m2) for 3 h, or HU (10 mM) for 8 h, mRNAs were extracted from HEK293T cells and subjected to qRT-PCR with the USP36 primers. The data shown are mean ± SD of three independent experiments. Statistical analyses were performed with the two-tailed Student's t-test. NS = not significant. (F) K48-ubiquitin chain type on USP36. USP36 stably knock-down HEK293T cells were transiently transfected with indicated HA-tagged ubiquitin and Flag-USP36. After 48 h, cells were treated with MG132 (50 μM) for 3 h before collecting. Cell lysates were subjected to immunoprecipitation with anti-Flag agarose beads, and then blotted with the indicated antibodies. (G) USP36 stably knocked-down HEK293T cells were transiently transfected with indicated constructs. After 48 h, cells were untreated or treated with HU (10 mM) for 8h. The cells were lysed and western blot was performed with the indicated antibodies.

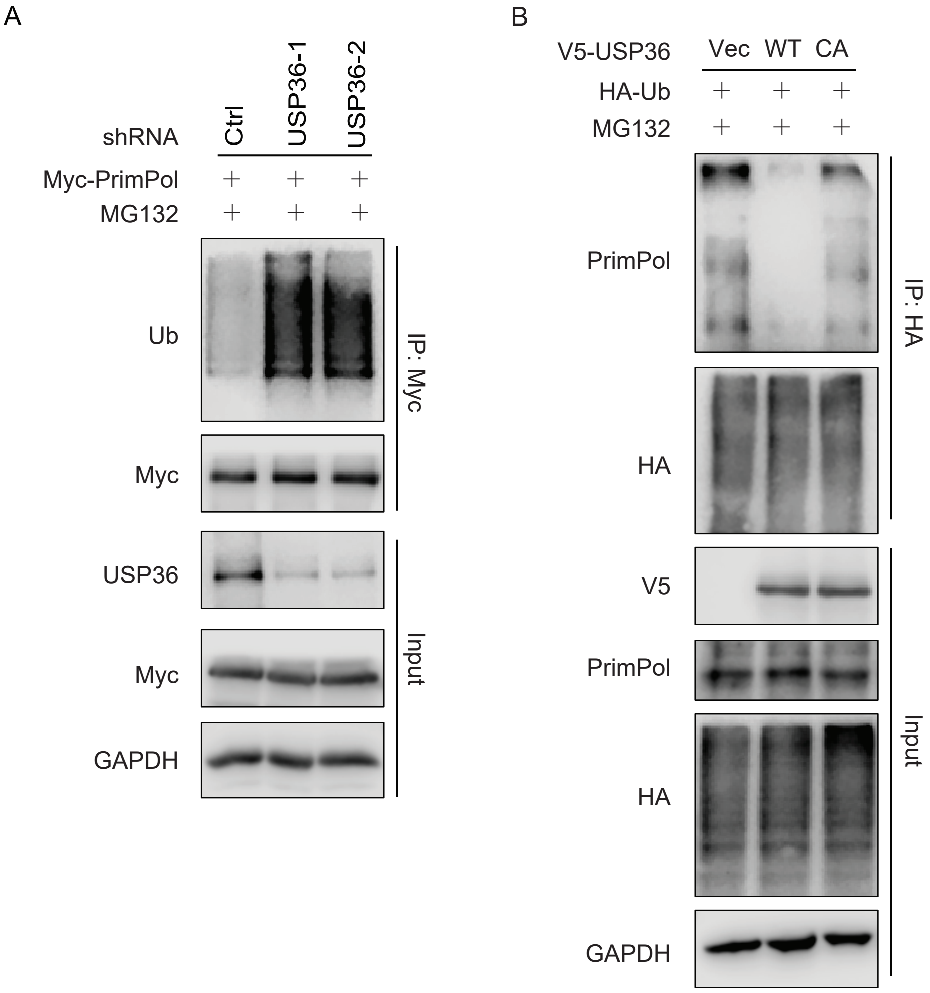
A
shRNA
Ctrl
USP36-1
USP36-2
Myc-PrimPol
MG132
Ub
Myc
USP36
Myc
GAPDH
IP: Myc
Input
B
V5-USP36
Vec
WT
CA
HA-Ub
MG132
PrimPol
HA
V5
PrimPol
HA
GAPDH
IP: HA
Input

Supplementary Figure S5. USP36 deubiquitinates PrimPol.

(A) OVCAR8 cells stably expressing control (Ctrl) or USP36 shRNAs were transiently transfected with Myc-tagged PrimPol. After 48 h, cells were treated with MG132 (50 μM) for 3 h. Cell lysates were subjected to immunoprecipitation with anti-Myc agarose beads, and then blotted with the indicated antibodies. (B) USP36-WT, but not USP36-CA mutant, resulted in reduced polyubiquitination of endogenous PrimPol. HEK293T cells with USP36 stably knocked-down were transiently transfected with indicated constructs. After 48 h, cells were treated with MG132 (50 μM) for 3 h. Cell lysates were subjected to immunoprecipitation with anti-HA agarose beads, and then blotted with the indicated antibodies.

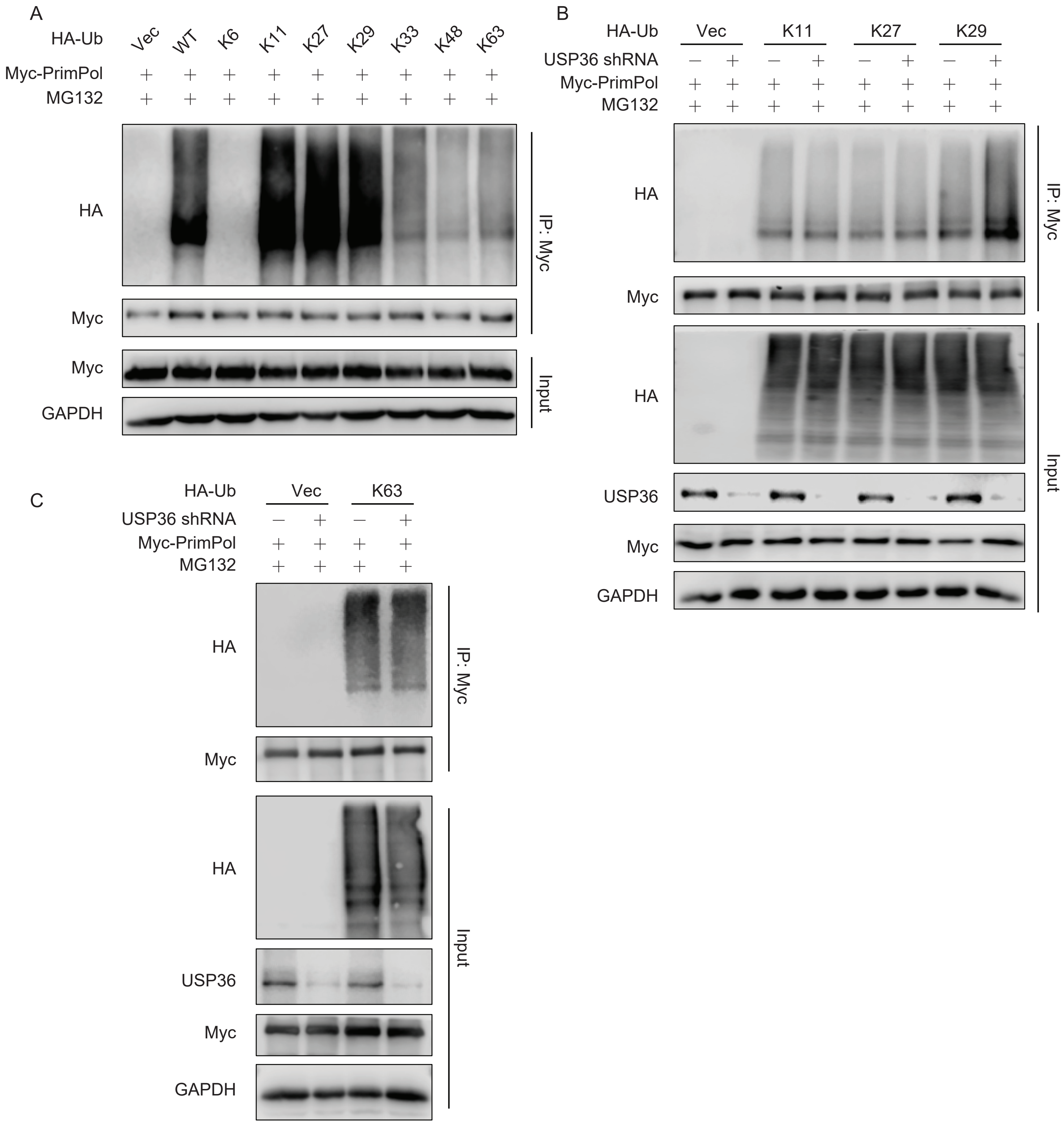
A
HA-Ub
Vec
WT
K6
K11
K27
K29
K33
K48
K63
Myc-PrimPol
MG132
HA
Myc
Myc
GAPDH
IP: Myc
Input
B
HA-Ub
Vec
K11
K27
K29
USP36 shRNA
Myc-PrimPol
MG132
HA
Myc
HA
USP36
Myc
GAPDH
IP: Myc
Input
C
HA-Ub
Vec
K63
USP36 shRNA
Myc-PrimPol
MG132
HA
Myc
HA
USP36
Myc
GAPDH
IP: Myc
Input

Supplementary Figure S6. USP36 regulates K29-linked polyubiquitination of PrimPol.

(A) PrimPol was ubiquitinated in the presence of Ub-K11, Ub-K27 and Ub-K29 *in vivo*. HEK293T cells with USP36 stably knock-down were transiently transfected with Myc-PrimPol and various Ub mutant plasmids. After 48 h, cells were treated with MG132 (50 μM) for 3 h, and a ubiquitination assay was performed. Cell lysates were subjected to immunoprecipitation with anti-Myc agarose beads, and then blotted with the indicated antibodies. (B) K29-linked polyubiquitination of PrimPol was largely induced in USP36 stably knockdown cells compared to control cells. HEK293T cells stably expressing control or USP36 shRNAs were transiently transfected with indicated HA-tagged ubiquitin and Myc-PrimPol. After 48 h, cells were treated with MG132 (50 μM) for 3 h before collecting. Cell lysates were subjected to immunoprecipitation with anti-Myc agarose beads, and then blotted with the indicated antibodies. (C) HEK293T cells stably expressing control or USP36 shRNAs were transiently transfected with HA-tagged Ub-K63 and Myc-PrimPol. After 48 h, cells were treated with MG132 (50 μM) for 3 h before collecting. Cell lysates were subjected to immunoprecipitation with anti-Myc agarose beads, and then blotted with the indicated antibodies.

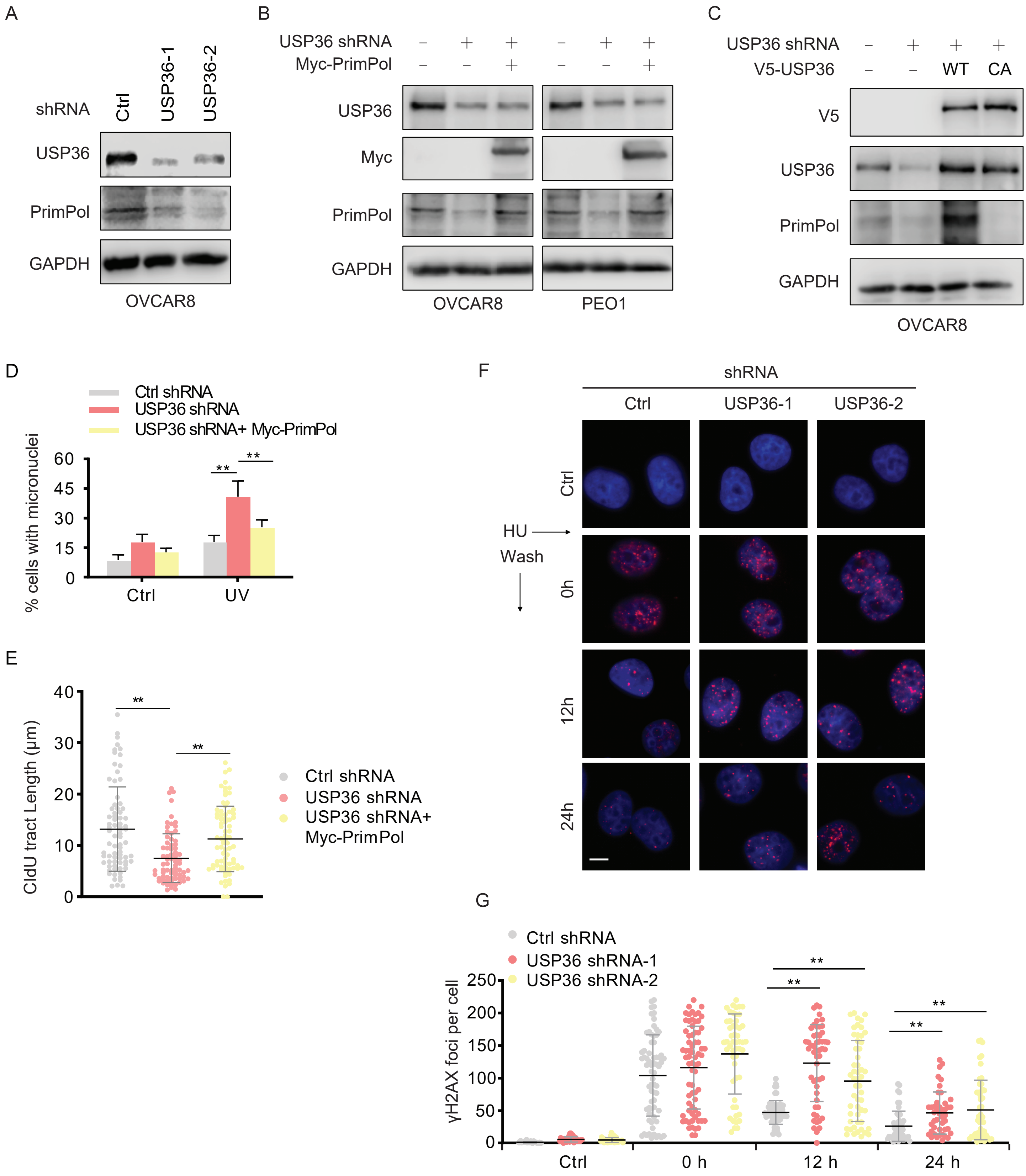

A
shRNA
Ctrl
USP36-1
USP36-2
USP36
PrimPol
GAPDH
OVCAR8
B
USP36 shRNA
Myc-PrimPol
USP36
Myc
PrimPol
GAPDH
OVCAR8
PEO1
C
USP36 shRNA
V5-USP36
WT
CA
V5
USP36
PrimPol
GAPDH
OVCAR8
D
Ctrl shRNA
USP36 shRNA
USP36 shRNA+ Myc-PrimPol
% cells with micronuclei
Ctrl
UV
E
CldU tract Length (µm)
Ctrl shRNA
USP36 shRNA
USP36 shRNA+ Myc-PrimPol
F
shRNA
Ctrl
USP36-1
USP36-2
HU
Wash
0h
12h
24h
G
Ctrl shRNA
USP36 shRNA-1
USP36 shRNA-2
γH2AX foci per cell
Ctrl
0 h
12 h
24 h

Supplementary Figure S7. USP36 participates DNA replication stress in a PrimPol-dependent manner.

(A) USP36 depletion reduces PrimPol protein level. OVCAR8 cells were transduced with lentivirus encoding control (Ctrl) or USP36 shRNAs, and cell lysates were blotted with indicated antibodies. (B) OVCAR8 and PEO1 cells stably expressing Ctrl or USP36 shRNAs were transiently transfected with the Myc-PrimPol. Cell lysates were then blotted with the indicated antibodies. (C) OVCAR8 cells stably expressing Ctrl or USP36 shRNA-2 were transiently transfected with the wild-type (WT) and catalytically inactive C131A mutant (CA) of V5-tagged USP36. The cells were lysed and western blot was performed with the indicated antibodies. (D) PEO1 USP36-knockdown cells reconstituted with Myc-PrimPol from Supplementary Figure S7B were used for measuring micronuclei. Cells were left untreated or treated with UV (5 J/m2) for 48 h. Cells were fixed, permeabilized in 0.5% Triton X-100, stained with DAPI, then analyzed by Nikon eclipse 80i fluorescence microscope for micronuclei. The micronuclei assays were repeated for 3 times, and at least 200 cells were counted for each experiment. (E) PEO1 USP36-knockdown cells reconstituted with Myc-PrimPol from Supplementary Figure S7B were used to detect the restart efficiency of stalled replication forks. Cells were labeled with IdU for 20 minutes as a control to label the active replication, and followed by incubation with 4 mM HU for 2 h. Cells were then labeled with CldU for 1h to evaluate the DNA replication upon genotoxic stress. DNA fibers were stretched on a microscope slide, stained with IdU and CldU antibodies, imaged, and the lengths of fiber tracks measured. (F) Representative images of γH2AX foci (red) in Ctrl and USP36 knock-down OVCAR8 cells treated with 5 mM HU for 12 h and released for indicated times. Nucleus is stained with DAPI (blue). Scale bar in the bottom left is 10 μm. (G) Quantification of γH2AX foci in OVCAR8 cells from Supplementary Figure S7F. The graphs represent mean ± SD, two-tailed, Student’s t-test. $*p < 0.05$; $**p < 0.01$.

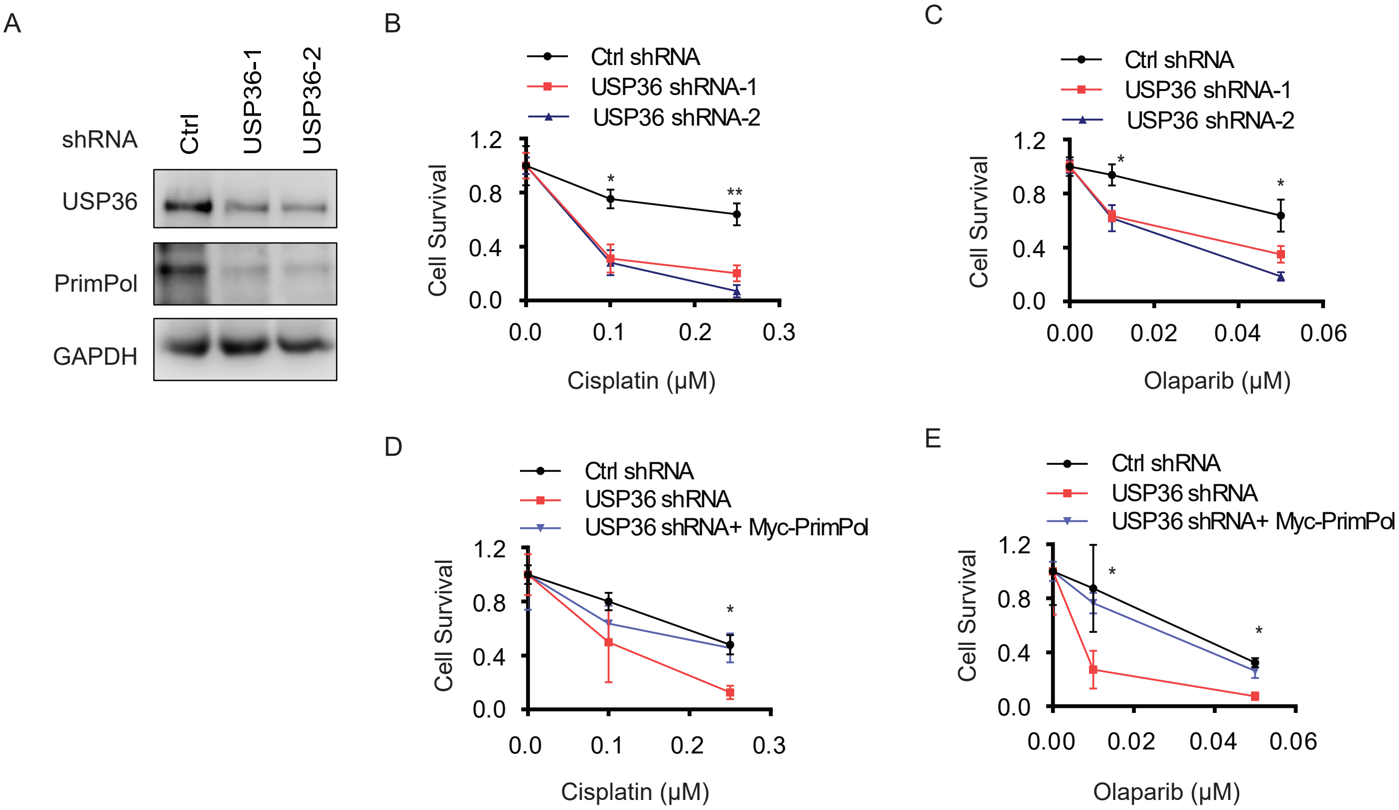

A
shRNA
Ctrl
USP36-1
USP36-2
USP36
PrimPol
GAPDH
B
Ctrl shRNA
USP36 shRNA-1
USP36 shRNA-2
Cell Survival
1.2
0.8
0.4
0.0
0.0
0.1
0.2
0.3
Cisplatin (μM)
*
**
C
Ctrl shRNA
USP36 shRNA-1
USP36 shRNA-2
Cell Survival
1.2
0.8
0.4
0.0
0.00
0.02
0.04
0.06
Olaparib (μM)
*
*
D
Ctrl shRNA
USP36 shRNA
USP36 shRNA+ Myc-PrimPol
Cell Survival
1.2
0.8
0.4
0.0
0.0
0.1
0.2
0.3
Cisplatin (μM)
*
E
Ctrl shRNA
USP36 shRNA
USP36 shRNA+ Myc-PrimPol
Cell Survival
1.2
0.8
0.4
0.0
0.00
0.02
0.04
0.06
Olaparib (μM)
*
*

Supplementary Figure S8. USP36 regulates the treatment response of cancer cells through PrimPol stabilization.

(A) USP36 depletion reduces PrimPol protein level. PEO1 cells were transduced with lentivirus encoding control (Ctrl) or USP36 shRNAs, and cell lysates were blotted with indicated antibodies. (B-C) Control and PEO1 cells with USP36 stably knock-down from Supplementary Figure S8A were subjected to cisplatin or olaparib treatment. Survival of the cells was assessed by colony formation assay. (D-E) PEO1 USP36-knockdown cells reconstituted with Myc-PrimPol from Supplementary Figure S7B were subjected to cisplatin or olaparib treatment. Survival of the cells was assessed by colony formation assay. Error bars represent ± SD from three independent experiments. $*p < 0.05$; $**p < 0.01$.
